# The Enigmatic Cytokine Oncostatin M and Roles in Disease

**DOI:** 10.1155/2013/512103

**Published:** 2013-12-08

**Authors:** Carl D. Richards

**Affiliations:** McMaster Immunology Research Centre, Department of Pathology and Molecular Medicine, McMaster University, 1280 Main Street, West, Hamilton, ON, Canada L8S 4K1

## Abstract

Oncostatin M is a secreted cytokine involved in homeostasis and in diseases involving chronic inflammation. It is a member of the gp130 family of cytokines that have pleiotropic functions in differentiation, cell proliferation, and hematopoetic, immunologic, and inflammatory networks. However, Oncostatin M also has activities novel to mediators of this cytokine family and others and may have fundamental roles in mechanisms of inflammation in pathology. Studies have explored Oncostatin M functions in cancer, bone metabolism, liver regeneration, and conditions with chronic inflammation including rheumatoid arthritis, lung and skin inflammatory disease, atherosclerosis, and cardiovascular disease. This paper will review Oncostatin M biology in a historical fashion and focus on its unique activities, *in vitro* and *in vivo*, that differentiate it from other cytokines and inspire further study or consideration in therapeutic approaches.

## 1. Introduction

Despite the original identification and cloning of the human cytokine Oncostatin M (OSM) in the late 1980s and an increasing interest in its study more recently, there is still a considerable amount that is not clear about its biology, function *in vivo,* and contradictory points of view on its role in certain conditions. The grouping of OSM into the family of gp130 (or IL-6/LIF) cytokines has been useful in rationalizing some redundant functions among this group and to explain lack of severe phenotypes in gene deficient mice. However, this grouping may also mask unique effects of OSM that has significant effects upon over- or underexpression in adult mammals. Reviews specifically on OSM have been published in 2003 and 2004 [1, 2] and other more recent reviews of the gp130 cytokine family including those of Silver and Hunter [3] and Sims and Walsh [4] incorporate aspects of OSM biology. This review will focus on OSM, discuss its activities relevant to pathology, and examine functions of OSM that are distinct from other gp130 family members, with a view to suggest further study into this interesting molecule and its role in disease.

## 2. Discovery, Cloning, and Expression

OSM was first purified and biochemically characterized on the basis of its antiproliferative activity on the A375 human melanoma cell line *in vitro* [5]. Its name was coined based on this inhibitory function on A375 and other melanoma cell lines. A potential function in the regulation of cancer was explored and further studies have clearly shown pleiotropic actions in hematopoietic, skeletal tissue alteration, metabolic, immunologic, differentiation, and inflammatory disease processes as outlined in more detail below. OSM was also found to interact with receptor complexes that included the cell signaling molecule gp130 and thus has been grouped with this gp130 family of cytokines in general.

The unique full length sequence of human OSM upon molecular cloning from U937 cells was completed in 1989 [6] to determine a 2 kb mRNA transcript encoding a 252 amino acid polypeptide with a hydrophobic N-terminus signal peptide and 3′AT-rich untranslated region similar to other cytokines. Although the full length form is active, a hydrophilic C-terminus region is cleaved off to yield a mature form of 196 amino acids with higher biological activity [7]. Cloning of the mouse OSM cDNA was completed in 1996 [8] and rat OSM cDNA has also been cloned [9]. The crystal structure of human OSM reveals a typical hematopoietic cytokine topology with up-up-down-down four-helix bundle with specific site 2 and site 3 epitopes predicted to interact with receptor chains on cell surfaces [10–12]. Human OSM has also been found to bind to collagens I, III, and IV *in vitro* [13] and, while OSM maintained its bioactivity in this context, this suggests that interstitial collagens are involved in its spatial pattern of bioavailability in tissues.

Individual cell populations that express OSM include activated macrophages, monocytes, T cells, and dendritic cells [5, 6, 14] (reviewed in [1]) although it is not clear if specific subsets of T cells differentially express OSM. Grenier et al. [15] showed that OSM could be released by neutrophils from preformed stores and these cells could also synthesize OSM upon stimulation by LPS or GM-CSF. OSM was also shown to be released by neutrophils from patients with acute lung injury [16]. Enhanced levels of OSM could be detected systemically in septic shock [17] and early (day 1) in patients with overt bacterial peritonitis [18]. Monocyte/macrophages, a primary source of OSM, release OSM upon stimulation with agents such as TLR-ligands or prostaglandins [19]. Generally OSM systemic levels are low in chronic inflammatory conditions and levels at local sites of inflammation are more indicative of potential function in chronic disease (see below).

OSM expression during mouse development was noted in hematopoietic cells of developing liver and later in bone marrow, thymus, and spleen by *in situ* hybridization [20]. OSM mRNA was also detected after birth in tissue eosinophils of small intestine, lung, and skin [20] and is expressed significantly in bone marrow in adult mice [8]. OSM expression was noted in IL-5R*α* subunit expressing cells [20], and its cloning as an IL-3/IL-5/GMCSR-induced immediate early gene in mice [8] implicates OSM involvement in hematopoiesis. In addition to neutrophil and eosinophils, mast cells have been shown to produce OSM upon T-cell activation but not upon IgE cross-linking [21].

## 3. OSM as a Member of the gp130 (IL-6/LIF) Family of Cytokines

The gp130 cytokines are proteins secreted by immune and nonimmune cells and function to modulate differentiation, hematopoietic, immune, and inflammatory cell networks. Several reviews provide extensive information regarding these molecules [22–26]. These cytokines are grouped together on the basis of the use of the signalling molecule gp130 as a subunit of the cell surface receptor complexes. Thus IL-6, leukemia inhibitor factor (LIF), interleukin-11 (IL-11), cardiotrophin-1 (CT-1), ciliary neurotrophic factor (CNTF), neurotrophin-1/BSF-3 [27], IL-27 [28], and OSM induce responses upon binding to receptor complexes which are dependent on gp130 and thus possess some redundant biological activities [10, 29–31]. IL-31 is another member of this family but engages a receptor complex that requires gp130-like protein (GPL) rather than gp130 [32].

### 3.1. Receptors

**Figure 1:**
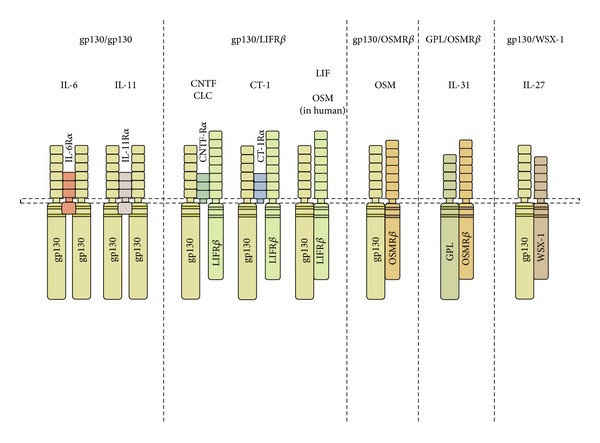
Schematic diagram of the gp130 cytokine receptor complexes. Members of the gp130 cytokine family engage in receptor complexes that include the gp130 signal transduction chain with the exception of IL-31, which engages a complex that utilizes gp130-like (GPL or IL31*α*) chain. The family may be subdivided further according to the signaling chains utilized: gp130/gp130; gp130/LIFR; gp130/OSMR*β*; GPL/OSMR*β*, and gp130/WSX-1, (modified from Fritz [39]).

Cellular responses to individual family members reflect the presence (or absence) of expression of the individual cytokine-specific receptor subunits on cell surfaces. Despite some overlapping biological activities, gp130 cytokines also display divergent functions *in vitro* and likely have both shared and unique functions *in vivo*. The ligands of the IL-6 family were used to characterize and clone the receptor chain complexes. The gp130 family of cytokines, receptors, and cell signaling has also been the subject of excellent reviews [23, 30, 33–35]. These describe IL-6, IL-11, CNTF, LIF CT-1, and IL-31, all binding to a more specific binding receptor chain (IL-6R*α*, IL-11R*α*, CNTFR, and LIFR*α*) which then recruits gp130 for higher affinity and cell signaling (see Figure 1). In contrast, OSM binds gp130 with low affinity as described by Liu et al. [36] and Gearing et al. [37] and as such has little to no biological activity unless a second receptor chain is recruited, either the LIFR*α* [37] or the specific OSMReceptor-beta chain (OSMR*β*) [38]. The gp130/LIFR*α* complex was termed as OSM receptor type I, and the OSMR*β*/gp130 complex termed as the OSM receptor type II in the human system.

This terminology later became less relevant for the mouse system since mouse OSM binds mouse OSMR*β*/gp130 but not the mouse LIFR*α*/gp130 complex. Sequencing and expression of mouse OSM enabled the identification and cloning of the mouse receptor homologue of OSMR*β* and the characterization that the mouse OSM ligand did not engage or signal through the mouse LIF receptor [40–42]. This clearly indicated functional dissimilarities in the cytokine biochemistry and predicted functional differences in its biology between species. Interestingly, the OSM receptor system in rats has more recently been characterized [43] and shows that rat OSM engages both the LIF receptor and the OSM specific receptor in this species, analogous to the human type I and type II receptor. As the authors note, this suggests that studies in rats of OSM may more closely resemble human OSM receptor function and biology *in vivo*.

### 3.2. Cell Signaling

Several signalling pathways (JAK/STAT3, MAP Kinase (MAPK), and PI3′Kinase (PI3′K) [44–48]) can be stimulated by gp130 cytokines, although the spectrum of pathways enlisted varies depending on the cell type and differential recruitment of signalling pathways. The STAT family of transcription factors has been established as critical cell signalling molecules involved in immunity and inflammation [49–51] and mediates IFN*α* and IFN*β* (STAT1 and 2), gp130 cytokines and IL-10 (STAT3), IL-12 (STAT4), prolactin and growth hormone (STAT5A and 5B, resp.), and IL-4/IL-13 (STAT6) signaling. STATs are latent transcription factors that are phosphorylated by the Janus family of tyrosine kinases (JAKs) upon ligand interaction with cell surface receptor complexes; translocate to the cell nucleus and bind to specific DNA sequences in promoters or to other transcription factors to modulate gene expression. Their activity can be further regulated through serine phosphorylation [52] and inhibited by intracellular SOCS (suppressors of cytokine signaling proteins) and PIAS (protein inhibitor of activated STAT) [53, 54].

PI3′K are a family of kinases that phosphorylate the 3′hydroxyl group of phosphatidylinositols, recruit, and facilitate activation of serine/threonine kinases including Akt (PKB) and PDK-1 which can regulate signalling pathways that influence cell survival and differentiation [55, 56]. PI3′K/Akt activity has been implicated in cellular processes that make up the hyperproliferative, aggressive cellular environment identified during chronic inflammatory disease [57]. MAPK pathways transmit the signals of a range of cytokines and growth factors to regulate a broad spectrum of cell phenotypic behaviour [58].

**Figure 2:**
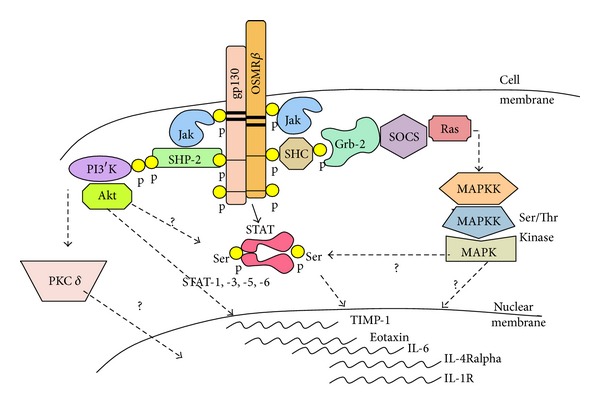
OSM receptors and signaling. A schematic representation of signal transduction initiated through the OSMR/gp130 receptor complex. While several gp130-utilizing cytokines activate JAK/STAT and MAPK signaling pathways in connective tissue cells, we observe that OSM uniquely activates additional signaling intermediates including STAT5, STAT6, the PI3′K/Akt pathway, and the novel PKC isoform PKC delta (PKCd) in fibroblasts. OSM induces IL-4Ralpha and IL-1R (modified from Fritz [39]).

On the basis of *in vitro* studies, the OSM ligand recruits a broader array of cell signaling pathways than most gp130 cytokines, likely on the basis of the extent and amount/cell of cellular expression of the OSMR*β* subunit. OSMR*β* and gp130 chains are widely expressed in connective tissue cells, and in bioassays OSM is consistently more active than other gp130 cytokines in regulating genes such as IL-6, MMP-1 and TIMP-1, hepatocyte acute phase proteins, and chemokines such as eotaxin-1 (see Figure 2).

Although OSM is most closely related to LIF [1] and engages the LIFR*α*/gp130 receptor complex, OSM ligand binding to its own specific (type II) receptor engages additional pathways highlighting nonredundant functions of the OSMR*β*/gp130 signaling complex. Auguste et al. [59] showed that JAK1, JAK2, and Tyk 2 were activated by LIF and OSM but that only OSM also activated STAT5b. Hermanns et al. [60] showed that OSM, but neither LIF nor IL-6, induced Shc isoforms and association with adaptor molecule Grb2 and also that Shc was recruited to OSMR*β* but not gp130. This was associated with stronger ERK1/2 MAPKinase activation, and later studies also showed strong and prolonged induction of SOCS3 by OSM compared to IL-6 signaling [61]. Focusing on mouse OSM and its receptor system, we have shown that OSM regulates STATs-1 and -3, as do other gp130 cytokines, but also induced STAT5, STAT6, Akt, and Protein Kinase C delta [62–64] activation in mouse fibroblasts. Collectively, the data supports unique signaling by OSMR*β*/gp130 pathways and predicts unique functions of OSM *in vivo*.

The identification and cloning of IL-31 [32] added an interesting complexity to the OSMR system since IL-31 receptor complex requires OSMR*β* and the specific GPL (IL-31R*α*) protein [65]. The apparent differences in function of IL-31 from OSM are striking in context of the sharing of the OSMR*β* receptor as a required partner in cell signalling. The intracellular signals generated by IL-31 are distinct from OSM in lung epithelial cells [66] and the level of expression of IL-31Ralpha (GPL) is also markedly lower in epithelial cells [67]. IL-31 is produced by activated T helper 2 cells and IL-31 transgenic animals showed skin lesions and alopecia [32]. Subsequent work showed that IL-31 and GPL expression were elevated in atopic dermatitis at the mRNA [68] and protein level [69]. On the basis of IL-31 knockout mouse responses, IL-31 was hypothesized to have a dampening effect on airway allergic inflammation induced in a mouse model of *Schistosoma mansoni* egg administration [70]. However, later studies showed that IL-31R*α* deficient mice have elevated responsiveness to OSM both *in vitro* and *in vivo* and the authors suggest that previously observed effects on IL-31Receptor knockout mice were due to elevated OSM biological function in lung [71].

Soluble forms of gp130 and OSMR*β* have been characterized and detected in normal human sera [72]. These may contribute to control of OSM activity by forming complexes with OSM, although transsignalling such as that seen by IL-6/solubleIL-6R or transsignalling inhibition by soluble gp130 [73, 74] has not thus far been identified. A synthetic OSMR*β*-gp130 fusion protein that has high affinity and inhibitory activity for mouse OSM [75] maybe a very useful tool.

## 4. Mouse Transgenic Studies, OSM, and OSMReceptor*β* Gene Deficiency

Effects of transgenic overexpression have suggested pathogenic roles for OSM. Earlier studies showed that mice transgenic for bovine OSM possessed bone abnormalities, fibrosis around the pancreas, and abnormal lymphoid tissue [76, 77]. Another study showed that OSM transgene induced increases in both mature and immature extrathymic T cells [78], and this was not dependent on thymus since nu/nu mice could be reconstituted with OSM-transgenic bone marrow cells to see the same effect. Transient overexpression systems of OSM using Ad vector to explore local tissue effects have been used to characterise effects in joint, skin, and lung [79] (more details below) and the data suggest that effects of overexpression can be qualitatively much different depending on the tissue/organ site.

There have been relatively few articles published on OSM ligand deficient mice. Morikawa et al. [80] have shown that during development, OSM knockout mice show decreases in neurons with nociceptive phenotype within the dorsal root ganglion, and mice showed reduced sensitivity to several different pain stimuli. This suggests a potential role in pain perception, although whether manipulation of OSM function after development of the central nervous system has similar effects would be an interesting question.

More recently the work of Esashi et al. shows the intriguing observation that OSM knockout animals have reduced thymocytes and abnormal thymus structure, associated with glomerulonephritis and autoantibody against dsDNA in aged mice [81]. The authors suggest that OSM plays a role in preventing clearance of apoptotic thymocytes that contributes to development of autoimmune disease. On the other hand, Clegg et al. [82] many years earlier showed increases in double stranded DNA antibodies in serum of mice transgenic for OSM under the control of the proximal lck promoter. This was associated with a decrease in the thymocytes, but with an increase in CD8 positive T cells. It is not clear how to rationalize similar effects on increasing autoantibody in both OSM knockout mice and OSM transgenic mice, other than speculating about the complexity of prolonged expression or underexpression through development or the cell type generating the overexpression system. That being noted, the involvement of OSM in autoimmune antibody generation is a potential area for further investigation.

Mice deficient in OSMR*β* chain have been generated and are useful for examining the function of the specific OSM receptor in the mouse. Tanaka et al. [83] showed that although the OSMR*β* knockout mice were healthy and fertile, hematopoietic alterations were evident including erythrocyte and platelet levels which were reduced compared to wild-type animals. Chimeric OSMR*β* knockout mice engrafted with wild-type bone marrow showed reduced erythrocytic and megakaryocytic progenitors, indicating that the OSMR*β* was required for the hematopoietic microenvironment. A number of other studies have shown defects of OSMR*β*-deficient mice in acute inflammation [84], liver regeneration [85], thymic hypoplasia [81], and net metabolism of bone [86] and fat [87].

## 5. Effects on Hepatocytes and Liver

The liver responds to inflammatory mediators with the synthesis of a number of acute phase proteins that are spilled into the systemic circulation and result in higher levels of these proteins back at the site of inflammatory stimulus (reviewed in [88]). The identification of OSM as a regulator of hepatocyte acute phase protein response in 1992 [89] helped to group the cytokine on the basis of functionality alongside IL-6 and LIF at that time. Published work has shown that OSM regulates LDLR in liver cells [90] suggesting a role in cholesterol metabolism. More recent studies have identified hepcidin as a target for regulation by OSM [91, 92] resulting in decreased serum iron levels and implicating OSM in disorders involving anaemia.

The potential role of OSM in mouse liver development was suggested by studies identifying its function in fetal liver hepatic cell differentiation [93] and fetal liver hematopoiesis. Nakamura et al. found that OSMR*β* knockout mice had impaired liver regeneration responses upon liver damage within models of carbon tetrachloride (CCL4) exposure or partial hepatectomy [85]. They also found that IL-6 deficiency failed to express OSM in response to CCl4, and subsequent administration of OSM induced liver cell cycle genes. Studies have gone on to investigate the function of OSM as a liver regenerating factor. Following work in 2005 that showed OSM-stimulated differentiation of rat oval cells toward hepatocytes [9], a gene therapeutic approach using OSM cDNA in rats showed an attenuation of liver damage following dimethylnitrosamine treatment [94]. Patent positions have been prepared in this arena, suggesting potential for a therapeutic approach to liver damage.

OSM is expressed by Kupffer cells, and along with other gp130 cytokines, regulates hepatocyte responses [89]. In liver fibrosis, hepatic stellate cells play an important role in fibrogenesis [95], and contributions by OSM have been suggested. For example, Znoyko et al. [96] examined cirrhotic and normal liver human samples and showed that although OSM was elevated in cirrhotic tissue, the OSMR*β* was absent, and the LIFR*α* was significantly increased. This might suggest that functioning OSM in this fibrotic system acts through the OSMReceptor type I (the LIF receptor complex in humans).

## 6. OSM Regulates Joint Synovium and Cartilage Remodeling

**Figure 3:**
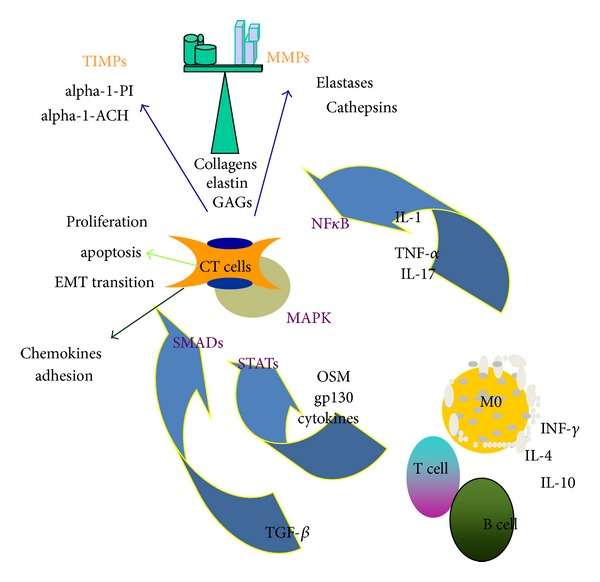
Schematic diagram of OSM and extracellular matrix remodeling. OSM and other gp130 cytokines are produced by macrophages and T cells and can be regulated by Th1 and Th2 cytokines. OSM, catabolic cytokines (IL-1, TNF, and IL-17), and fibrogenic TGF*β* are potent regulators of connective tissue cells (CT cells, which here include fibroblasts, chondrocytes, osteoblasts, smooth muscle cells, and epithelial cells) through cell signaling molecules STATs, SMADs, NFkB, and MapKinases (shown in purple). Products expressed include matrix metalloproteinases (MMPs), their inhibitors (TIMPs) other enzymes, and components of the ECM. CT cells also express chemokines and adhesion molecules that contribute to inflammatory processes and can be regulated through proliferative, apoptotic, and EMT transition to alter tissue pathology in inflammatory diseases.

The initial observations that IL-6 [97] and later OSM [98–100] were present in the synovial fluid of rheumatoid arthritis patients stimulated the study of their potential roles in inflammatory joint disease. Subsequent to observations of the regulation of TIMPs *in vitro* [101] OSM was shown to regulate the balance of matrix metalloproteinase (MMP) and their inhibitors (TIMPs) in connective tissue cells. Functions of OSM contribute to the network of cytokines, mediators, enzymes, and structural proteins that control net ECM (see Figure 3). *In vitro*, OSM regulates TIMP-1 and TIMP-3 [102] in chondrocytes and was originally thought to contribute to a protective role in extracellular matrix (ECM) catabolism. However, studies *in vitro* showed that mouse OSM induced MCP-1/CCL-2 production and anchorage independent growth of mouse synovial cells [103] and human OSM induced CCL-13 in human synovial fibroblasts [104] indicating a potential proinflammatory role.

### 6.1. Cartilage Catabolism

Furthermore, based on studies of cartilage *ex vivo*, a catabolic function for OSM in the regulation of chondrocytes and their matrix became clear. The work by the group of Cawston et al. has shown robust activity of OSM in combination with established proarthritis mediators [98, 105] through the upregulation of the matrix metalloproteinase (MMP) expression and enzymatic activity. Thus OSM in combination with IL-1*α* caused maximal collagen release from cartilage explants of either bovine or human origin [105, 106]. Examination of the genes regulated by OSM/IL-1*α* showed synergistic regulation of several MMPs human chondrocyte cultures *in vitro*, including MMP-1, 3, 8, 13, and 14 [107, 108]. Of significance in the cartilage explant studies was that, among the gp130 cytokines tested, only OSM, or IL-6 if the soluble IL-6Receptor was also added to the culture system, induced this synergistic set of responses [106]. Furthermore, IL-17A-induced bovine cartilage catabolism was also synergistically enhanced by OSM [109] where degradation was inhibited with a synthetic metalloproteinase inhibitor.

Consistent with these *in vitro* observations, models of mouse arthritis showed ameliorated joint inflammation when treated with an antibody to OSM [110], encouraging further work on OSM as a potential target for inflammatory joint disease. This was in stark contrast to previous work in 1999 [111] suggesting that the systemic administration of human OSM ligand to animal models of arthritis was protective. Wallace et al. [112] had shown that injection of human recombinant OSM reduced LPS-induced TNF*α* levels and lethality. They also showed marked reduction in inflammatory parameters in the anticollagen antibody cocktail-induced model of RA in C57Bl6 mice, administering human OSM (iv, daily for 10 days). Systemic human OSM in this study also ameliorated inflammation and damage in an EAE model. However, it later became clear that the interpretation of experiments using human OSM in mouse models was compromised by the species specificity within the biology of OSM, in that human OSM does not engage the specific mouse OSM receptor (see above). The anti-inflammatory effects likely engaged the mouse LIF receptor or possibly other undefined receptors to generate the observations of anti-inflammatory functions in mice.

### 6.2. Overexpression of OSM in Joints *In Vivo*

Overexpression studies in transgenic animals using either human or bovine OSM cDNA [77] could also be subject to potential problems with interpretation due to the species specificity. Bamber et al. [77] and Clegg et al. [113] assessed mouse OSM transgenic effects but mainly focused on lymphopoiesis and autoantibody levels as noted above. Juan et al. [114] compared the effects of transgenic human-, bovine-, and mouse-OSM overexpression in female (C57BL/6J xDBA/2J) mice using retroviral constructs and showed far more mild effects in bone remodeling with mouse OSM. We and others have studied the effects of transient gene transduction using Adenovirus (Ad) vectors to express species-homologous cytokines in mice. These can be administered to achieve local site expression of transgene (such as joint, lung, or skin) over 2–12 days (depending on the vector and the site) after which time the vector is cleared and the transgene expression wanes. By administering Ad vectors locally to joint spaces in mice, a number of studies have investigated the effects of specific transgenes in mouse models of inflammatory joint inflammation. Using this approach, IL-4 and IL-10 overexpression can alter inflammatory parameters in the collagen-induced arthritis mouse model [115, 116].

Transient local overexpression of OSM using Ad vector induced synovial inflammation, pannus formation, and cartilage degradation upon intra-articular (ia) injection [103, 117]. Concomitant addition of OSM and either IL-1 or TNF*α* using Ad vector ia administration showed marked elevation of joint and bone remodeling with altered balance of MMP and TIMP in mouse cartilage [118–120]. This was consistent with the synergy seen in ECM modulation on the basis of *in vitro* analysis. Further studies on cartilage catabolism mechanisms using combinations of OSM/IL-1 or OSM/TNF cocktail as a model stimulus *in vitro* have shown induction of MMP-10 in chondrocytes and synovial fibroblasts [121], induction of fibroblast activation protein-*α* in chondrocytes [122], or that PI3′K/Akt [123] and protein kinase R [124] signaling may be potential targets to regulate synergistic cartilage catabolic effects generated by these cytokines.

Collectively, data suggest that OSM is capable of priming and inducing maximal responses to IL-1, TNF*α*, or IL-17A in arthritis and thus joint destruction. Data from models of arthritis performed in OSM knockout mice are not published or easily accessible at this time; however, there has been reasonable rationale to develop antibodies to target OSM for indications such as RA. Targeting the gp130 cytokine pathway with antibody to the IL-6R*α* subunit has shown efficacy in clinical trials in RA patients [97, 125]; however, lack of full clinical response in many patients as well as potential side effects suggests that additional approaches to TNF*α* or IL-6 blockade should be considered. The species specificity of the OSM responses highlights potential difficulties in the field in developing interpretable preclinical models in mice. Since the receptor expression is in hematopoietic as well as nonhematopoietic cells, humanized nu/nu or chimeric mice would possess both human and mouse OSM specific receptors that are species specific in their binding, as well as human and mouse LIF receptors that are not necessarily species specific.

## 7. OSM and Bone Metabolism

It has been established (reviewed in [4, 126]) that gp130 cytokines can regulate both osteoblast and osteoclast activity and thus bone metabolism. The notion that OSM could alter bone tissue was suggested first by the transgenic overexpression studies using OSM in mice that showed signs of osteopetrosis [76] depending on the promoter construct in which the transgene was expressed. Developmental abnormalities in spermatogenesis, keratinized epithelia, neurotoxicity, and lymphoid tissue were also observed.

### 7.1. Osteoblastogenesis

Work by Jay et al. [127] in 1996 showed biological activity of OSM and LIF in neonatal murine rat and mouse calvarial cell cultures, inducing proliferation and collagen synthesis but reducing alkaline phosphatase and bone resorption. However, human recombinant OSM was used for these studies, which does not engage the specific mouse OSMReceptor as noted above. Analysis of cell types responding to OSM in context of other gp130 cytokines was completed by Bellido et al. who showed expression of the type II OSM receptor on the mouse MG-63 osteoblast cell line [128] and also noted differences in levels of receptors for gp130 cytokines among the various lines used. The effects of OSM and gp130 cytokines likely depend on the stage of bone progenitor cells which may differentially express sufficient receptors, since IL-6/solubleIL-6receptor, or IL-11/solubleIL-11R, could induce osteoblast markers in murine embryonic fibroblasts, whereas OSM or CT-1 could not [129].

Subsequent studies indicated that both LIF and OSM could regulate rat calvarial cell osteoblast differentiation processes, where OSMR*β* was highly expressed early in osteoblastogenesis but plateaued or decreased later in the culture system [130]. The same group went on to show that using the same rat calvarial bone nodule assay system, the presence of mouse OSM (but not LIF, IL-11, or IL-6/sIL-6R) early in the cultures (days 1–3) increased bone colony numbers [131] but also may have had apoptotic effects in the rat calvarial cell culture systems. Thus the regulation of bone formation in assays *in vitro* appears to be sensitive to time-dependent presence of gp130 cytokines including OSM.

More recent studies using the species homologous cytokine and cell system more accurately reflect function of OSM and gp130 cytokines *in vitro* and *in vivo*.

Overexpression of mouse OSM upon intra-articular administration of Ad-mOSM in mouse knees using Adenovirus vectors induced bone apposition [132] along the periosteum *in vivo*, accompanied by the marked upregulation of cartilage catabolism in the synovial joint and growth plate damage [117]. The effect of mouse OSM *in vitro* on murine preosteoblastic C2C12 cells showed increases in alkaline phosphatase upon combination with bone morphogenic protein (BMP)-2 [132], suggesting recruitment of BMPs or other factors in OSM-induced effects on periosteal bone formation *in vivo*. The site of OSM overexpression very much affects the observations *in vivo*. Administration of Ad vector expressing mouse OSM into the tibia bone marrow of mice showed marked increase in bone apposition without any signs of bone resorption [133]. More recently, Walker et al. [86] have observed an osteopetrotic phenotype in mice lacking OSMR*β*. This is in contrast to expectations on the basis of osteopetrotic phenotype of bovine OSM transgenic mice, with the proviso that the LIFReceptor was likely engaged in that system. Indeed, Walker et al. [86] suggest that mouse OSM can regulate OSMR-/-osteoblasts and postulate this acts through the mouse LIFR, in contrast to the generally accepted notion that such an interaction is not functional in mice. Also proposed was that OSM/OSMR*β* interaction was responsible for osteoclast differentiation in their system. In a follow-up study this group provides evidence, using wild-type and OSMR*β* deficient mice, that OSM participates in the anabolic effects of parathyroid hormone [134] and thus may be integrated mechanistically into PTH standard therapy for osteoporosis in humans.

Studies done in human cell culture systems showed that conditioned medium of LPS-stimulated human monocyte/macrophages could induce differentiation of osteoblasts from human mesenchymal stem cells (MSC) [133], and antibodies to OSM or to LIF partially inhibited supernatant activities in osteogenesis. Antibodies to both OSM and LIF completed abrogated the effect. Knockdown by siRNA of receptor subunits of OSMR*β*, gp130, or STAT3 also inhibited these effects [133], substantiating the role of OSM and its specific type II receptor in human osteoblastogenesis. This is further supported by recent work showing that cord-blood-derived macrophages released OSM that enhanced MSC differentiation toward osteoblasts [135], and another study that showed monocyte contact with MSC elevated OSM expression and induced osteoblast phenotype from MSC in a STAT-3 dependent manner [136]. In addition to differentiation processes, OSM can regulate chemokine expression such as MCP-1/CCL-2 in osteoblasts [137] and thus may further contribute to cell accumulation in inflammatory arthritis through this pathway.

### 7.2. Osteoclastogenesis

It is well established that bone metabolism is a net balance of osteoblast activity and osteoclast acitivity. Osteoclastogenesis is also regulated by gp130 cytokines, and this contributes to a complex regulation of net bone metabolism by cytokines and growth factors. In the mouse coculture system using calvaria osteoblasts and bone marrow as source of osteoclast precursors, mOSM markedly increased osteoclast phenotypes (TRAP+) cells as did mouse LIF and mouse CT-1, whereas IL-6 did not (without the sIL-6R present) [138]. The addition of glucocorticosteroid synergistically enhanced the effects on osteoclastogenesis and may in part be due to the regulation of receptor chains for gp130 cytokines *in vitro*. Palmqvist et al. [139] showed that mouse calvarial bone explants responded with mineral and collagen release when stimulated with mouse OSM, human OSM, or LIF and regulated the expression of RANK and osteoprotegerin (OPG) in this system. The addition of OPG could inhibit the bone resorbing activity, suggesting a mediation through the RANK/RANKL in mouse calvarial bone.

### 7.3. Adipogenesis

The function of OSM in stimulating differentiation from multipotential mesenchymal precursor cells clearly shows it can inhibit adipogenesis while simultaneously stimulating osteoblastogenesis (as do other gp130 cytokines). One of the original observations regarding family member IL-11 biological activity was as an adipose inhibitory factor [140–142] and also as an inducer of osteoblastogenesis [129]. It became clear that several gp130 cytokines alter differentiation pathways of multipotential mesenchymal cells, switching development toward osteoblastogenesis and inhibiting pathways to adipocytes. OSM could skew osteoblasts from adipocytes in bone marrow stromal cell lines (e.g., TBR341-2) or skew skeletal muscle differentiation from smooth muscle cells (e.g., TBRB) [143]. In human adipose-derived mesenchymal stem cells, OSM induced bone markers Runx2 and osteocalcin, while inhibiting adipocyte markers PPAR*γ* and lipoprotein lipase [144].

Oncostatin M inhibited the adipocyte differentiation of 3T3 preadipocytes most strongly among gp130 cytokines and through ERK and STAT5 signalling pathways [145]. OSM, LIF, and CT can downregulate the LIFR thus incorporating cross-talk and inhibition between these cytokines [146]. The very recent identification of the OSMR*β* deficient mouse with increased adipose tissue upon aging, as well as glucose intolerance [87], paints a complex picture of the function of OSM and balance of bone and adipose upon aging in mice. Studies on OSMR*β* deficient mice suggest that long term lack of OSM signalling induces an osteopetrotic bone phenotype [86]; however, long term decrease on OSMR*β* would presumably reduce both OSM and IL-31 signalling, and this may complicate the interpretation of effects in aging mice. Publications regarding the assessment of IL-31 in bone metabolism or on osteoclasts and osteoblasts are scarce at this time. Interestingly, serum OSM levels were shown to be inversely related to age in a study on human patients by Hamilton et al. [147], the significance of which is not clear presently.

## 8. Oncostatin M and Cancer

The originally described activity of OSM in inhibiting the proliferation of melanoma cell lines A375 cells and SK-MEL-28 as well as lung A549 carcinoma cells and HTB10 neuroblastoma cells *in vitro* [5] generated interest in consideration of OSM as an anticancer agent. This potential was explored for a while, concomitant with studies by researchers at Bristol-Myers Research Institute in examining anti-inflammatory properties of human OSM in mouse models of arthritis and EAE [148]. As discussed previously, the anti-inflammatory effects could not be attributed specifically to mouse OSM receptor activation. Preclinical studies of human OSM for cancer treatment have not been completed, possibly due to side effects of systemically administered protein. It was shown in 1993 that OSM stimulates growth of Kaposis's sarcoma cells [149] and that it could activate the PI3′K [44] Stat-1 and -3, ERK, and JNK pathways [45] in these cells *in vitro*. In the last 10 or so years, functions of OSM have been further explored in various different cancer and tumour systems.

### 8.1. Melanoma

Although the growth of human melanoma cell lines was inhibited by OSM in several studies, metastatic melanoma cell lines lack responsiveness to OSM and this was correlated to the loss of the specific OSMR*β* chain [150]. Experiments by these investigators showed that histone deacetylase agents enhanced OSMR*β* expression and responsiveness of the cell lines, suggesting that epigenetic mechanisms alter the nature of metastatic melanoma *in vivo*. Another study on the 1286 melanoma cell line showed high SOCS-3 expression [151], the natural inhibitor of JAK/SAT3 signalling, and that forced suppression of SOCS-3 could render the cells again responsive to inhibitory effects of OSM or IL-6. In examining human stage 3 melanoma patient populations retrospectively, Lacreusette et al. [152] found that relapse-free survival correlated to sensitivity to OSM or IL-6 antiproliferative effects but not to responses to other cytokines including IFN*γ* or TNF*α*. In further examining molecular mechanisms, some of the patient cell lines showed defects in signalling pathways including PKCdelta-dependent activation of serine phosphorylation (ser 727) on STAT-3, which was required for OSM inhibitory activity [153]. Thus, there are various mechanisms by which melanoma cells may escape inhibition by OSM or IL-6.

### 8.2. Osteosarcoma

The regulation of osteosarcoma by gp130 cytokines and other agents has been studied from several aspects (reviewed in [154]). Chipoy et al. found that OSM could regulate rat osteosarcoma cell proliferation [155] and reduced osteoblast markers in differentiated osteosarcoma cells as well as mature osteoblasts but not in early mesenchymal progenitors. Further studies showed that OSM could sensitize osteosarcoma cells to the effects of apoptosis inducing agents such as staurosporine [156, 157] through a mechanism involving STAT5 and p53. The efficacy of the related apoptotic-inducing agent Midostaurin *in vivo* was enhanced upon systemic upregulation of OSM by adenoviral overexpression of OSM in rats [158]. Somewhat in contrast, Fossey et al. [159] showed in both canine and human osteosarcoma systems *in vitro* that OSM induced STAT3 mediated MMP-2 and VEGF expression and invasiveness through matrigel, supporting an invasive phenotypic alteration. This difference could reflect *in vitro* versus *in vivo* effects or complexities in receptor usage by mouse OSM in rats.

### 8.3. Breast Cancer

Douglas et al. (1997) [160] showed that receptor chains for gp130 cytokines were present in a majority of breast cancer cell lines suggesting that these cytokines could regulate these cells' function. OSM could inhibit the proliferation of 3/4 of the cell lines tested in that study, and IL-11, LIF, or IL-6 could inhibit proliferation but in a smaller proportion of the cell lines studied.

In using immunohistochemistry and Western blots analysis, higher percentages of positive samples for OSM, LIF, and OSMR*β* were seen in breast carcinoma and infiltrating tumours than in benign disease [161]. A recent study has shown that elevated OSMR*β* levels were associated with a shorter recurrence-free and overall survival times [162]. Studies on the role of OSM in breast cancer have shown that a complex set of activities are likely involved.

OSM inhibits proliferation while increasing S100A9 expression [163] in MCF-7 breast cancer cells. S100A7 (Psoriasin) has also been shown to be induced by OSM in breast cancer cell lines [164]. Despite being able to inhibit proliferation in murine adenocarcinoma M6 and metastatic M6c cells, as observed in other cell breast cancer lines, Holzer et al. [165] showed that OSM increased M6 cell-cell and cell-substrate attachment and *in vitro* assays of invasive capabilities. Jorcyk et al. later showed similar actions in two different human breast cell lines [166], indicating consistency of observations in human and mouse systems. Snyder et al. have shown, using both mouse and human systems, that OSM induces fascin through a STAT3-dependent mechanism and thus contributes to a migratory and invasive phenotype of breast cancer cells [167]. Furthermore, more recent data suggests that c-myc serves as a switch to alter breast cancer cell responses to OSM, where c-myc elevation reduces its antiproliferative effects and elevates anchorage independent growth, indicative of a metastatic phenotype [168]. Indeed, while OSM has inhibitory effects on osteosarcoma and chondrocarcoma [169], its growth stimulatory effect on Ewing sarcoma was associated with elevated c-myc [170]. In addition, OSM can induce epithelial-to-mesenchymal transition (EMT) and cancer stem cell-like features in breast cancer cells *in vitro* [171, 172]. Collectively, these data indicate a battery of direct effects of OSM on breast cancer cells.

### 8.4. Other Cancers

A protumourigenic role in other cancer types has also been suggested. Rather than inhibiting proliferation, OSM stimulates the proliferation of SKOV3 ovarian cancer cells [173] and Ewing sarcoma cell lines [170]. With respect to cervical cancer, OSMR*β* mRNA was elevated in cervical squamous cell carcinoma cells but not in squamous intraepithelial lesions [174]. The authors suggest that increase in the receptor expression was associated with late stages of cervical carcinogenesis and adversely with patient overall survival. A further study associated enhanced OSMR expression on cervical carcinoma cells with higher induction VEGF-A and endothelial tube formation *in vitro*, indicating angiogenic action [175]. Cell signaling upon OSM stimulation of tumour cells derived from neural tissue can also be detected, although responses vary, in that glioma cell line proliferation was not affected whereas OSM suppressed the proliferation of astroglioma cells [176, 177].

Both OSM and IL-6 could stimulate the proliferation of prostate cancer 22Rv1 cells [178] and stimulate u-Plasminogen Activator and VEGF in DU-145 prostate cancer cells [179]. In addition, OSM, along with LIF and IL-6, was detectable by immunohistochemistry in benign prostatic hyperplasia but expressed at increased levels in higher grade prostatic carcinoma [180]. Recently, Smith et al. [181] described data in mice to show *in vivo* that OSM and IL-6 paracrine secretion could enhance malignant progression of prostatic intraepithelial neoplasia that was generated by loss of PTEN function and thus PI3′K/Akt pathway over-activation.

Lung cancer is particularly difficult to treat and results in by far the highest mortality rate among the major cancers. Epithelial cells are a major source of lung carcinomas although OSM and other gp130 cytokines generally inhibit proliferation of normal lung epithelial cells. However, OSM had less inhibitory action on premalignant epithelial cell lines, suggesting that epithelial-to-mesenchymal transition (EMT) alters responses to OSM and other cytokines [182]. The observation of EMT induction by OSM or hepatocyte growth factor was noted in lung cancer or pancreatic cancer cell lines *in vitro* [183], indicating that direct regulation of cancer cells by OSM can be enhanced by other factors. On the other hand, and interestingly, Wang et al. [184] have shown that, in a mouse xenograft system, factors produced by human mesenchymal stem cells could induce mesenchymal-to-epithelial transition (MET) of human lung adenocarcinoma cells, and antibody to OSM was able to reverse the inhibitory effect, suggesting a protective role of OSM.

Chen et al. [185] have noted that human lung adenocarcinoma cells mainly expressed a short form of the OSMR*β*, which acts as a decoy receptor and thus decreased sensitivity to inhibitory effects of OSM on proliferation. The higher expression of this short form correlated with disease progression and poor prognosis. Furthermore, the OSMR*β* was found to be frequently methylated in primary colon cancer tissues but not in normal tissues, and the methylation resulted in silencing of the receptor and decreased sensitivity to OSM inhibition of proliferation [186–188]. A spliced variant encoding a soluble form of OSMR*β* was evident in esophageal cancer tissue but not normal tissues [189] and OSM receptor gene polymorphisms were described in a papillary thyroid cancer population [190]. Collectively, these works suggest that in digestive system cancers studied thus far, OSMR*β* protein functions in tumour cells are downregulated, desensitizing them to a putative antiproliferative role of OSM.

### 8.5. OSM and the Tumour Environment

The growth of many solid tumours is crucially dependent on noncancerous stromal/inflammatory cells within the tumour mass or surrounding it. Cancer and inflammation are closely intertwined, and it is clear that invasion of normal tissue by malignancy generates a host inflammatory response to solid tissue tumours. Tumors that successfully expand must manipulate or navigate host responses. Both angiogenesis and ECM remodeling are required in the growth and structure and metastatic potential of solid tumors. A significant body of literature supports the implication of hematopoietic-derived tumour associated macrophages (TAMS) and tumour associated neutrophils (TANS) in contributing to the tumour growth (as reviewed in [191]). The activity of such tumour associated nontumour “host” cells is clearly implicated in tumour progression, where cancer cells can benefit from normal organ repair mechanisms such as tissue remodeling and angiogenesis in order to expand. In addition to TAMs and TANs, the presence of altered fibroblast populations (cancer associated fibroblasts, CAFs) may also be crucial to significant tumour expansion [192, 193].

Since OSM and other gp130 cytokines participate in inflammatory mechanisms, their role in regulating tumour stromal cells in cancer progression is important to determine. In this context, the work by Queen et al. is interesting in that human neutrophils secrete Oncostatin M upon coculture with MDA-MB-231 or T47D human breast cancer cells [194]. A recent study has shown that breast cancer cell products can induce OSM from both monocytes and macrophages and that OSM cooperated with heparin-binding EGF-like growth factor (HB-EGF) to modulate tumour cell chemotaxis [195]. Recent work using the 4T1.2 mouse mammary tumor model showed that knockdown of OSM in 4T1.2 cells rendered them significantly less capable of establishing metastasis in bone, decreased osteoclastogenic action, and somewhat increased time of survival in the tumour resection model [196]. Thus breast tumour cell expression of OSM and modulation of nontumour stromal cells may recruit OSM into the cell interactions *in vivo* via TAMs or TANs or other cells such as osteoclasts. This suggestion is supported by recent data published by Benson et al. [197] who showed in a mouse model of pancreatic tumour metastasis to liver that OSM was localized by immuno-fluorescence to TAMs, but not TANs, in the tumour tissue. Although not known at this time, it would seem quite likely that OSM may also be involved in regulation of CAF phenotypes.

## 9. OSM and the Central Nervous System

Various studies have explored the functions of OSM in cells of the central nervous system (CNS) as previously reviewed [2] and its potential role in CNS inflammatory diseases. Detection of OSM by immunohistochemistry in brain tissues of multiple sclerosis patients showed localization to microglia, reactive astrocytes, and infiltrating leukocytes, whereas staining was not found in the parenchyma of normal brains [198]. Cerebral endothelial cell expression of MCP-1 and IL-6 are targets of OSM stimulation *in vitro*, as are elevations in astrocytes of COX-2, IL-6, VEGF, and PGE2 levels [199–201]. Astrocytoma gene responses to OSM include those involved in matrix remodelling and angiogenesis [202]. Glezer and Rivest [203] show that OSM enhances oligodendrocyte precursor activity which may function in tissue repair.

OSM activities upon exogenous intraventricular administration in mice include suppression of neural precursor cells (NPC) [204] in subventricular zones, consistent with *in vitro* suppression of NPC neurosphere generation by OSM but not LIF or CNTF. This is also consistent with these author's observations that OSMR*β* deficient mice have markedly more neural precursor cells in the brain subventricular zone [204]. Thus, OSM and OSMR*β* may be important for CNS homeostasis, as also implied by the sensory defects in the OSM knockout mice discussed above [80].

## 10. Lung, Inflammation, and Extracellular Matrix

Documentation of altered levels of OSM in human airway disease has been published over the past several years. In 2005, Kang et al. showed that, in examining OSM mRNA and OSM protein, levels were higher in samples of nasal mucosa of patients with allergic rhinitis compared to that of normal subjects [205]. Later, Simpson et al. [206] detected elevated levels of OSM protein in human sputum samples from asthma patients with severe airflow obstruction. Mozaffarian et al. detected elevated levels of OSM in the bronchoalveolar fluid of patients with idiopathic pulmonary fibrosis (IPF) [207] and increased expression of OSM in scleroderma-associated interstitial lung disease [208]. OSM has also been detected in bronchoalveolar lavage mast cells of patients with sarcoidosis [21]. Furthermore, Baines et al. [209] have shown data indicating samples of induced sputum from adult patients with chronic-obstructive pulmonary disease (COPD) (*n* = 22) showed higher OSM levels than healthy controls (*n* = 29) as assessed by ELISA. In addition, OSM mRNA was elevated in COPD sputum cells as assessed by RT-PCR. These clinical studies validate the investigation of the role of OSM in these various lung conditions.

As in other chronic inflammatory diseases, the IL-6/gp130 cytokines have been implicated in inflammatory lung disease and are clearly accompanied by many cellular functions and interactions that are regulated by multiple factors. The role of cytokines has been reviewed extensively in other excellent reviews on allergic airway disease [210, 211] and fibrotic lung disease [212–214], the former of which has clearly been associated with Thelper 2 type cytokines including IL-4, IL-5, and IL-13 in mouse models and in atopic human asthma. Increases in fibrotic reactions are associated with severe asthma, particularly subepithelial collagen deposition which contributes to decline of lung function and gas exchange.

**Figure 4:**
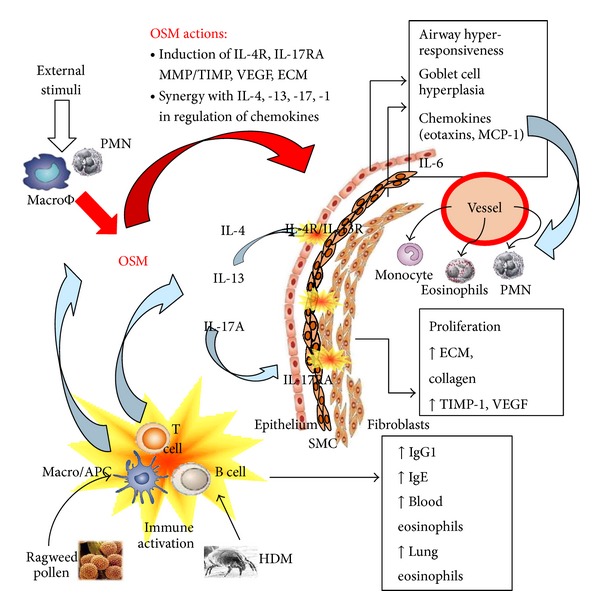
Postulated roles of OSM in allergen induced airway inflammation. Repeated exposure of allergens (Ragweed or House Dust Mite (HDM)) in airways leads to activation of the adaptive immune response resulting in secretion of Th2-associated immunoglobulins (IgE and IgG1) and cytokines (IL-4/IL-13). Local production of OSM by activated inflammatory cells regulates various responses synergistically in combination with IL-4, IL-13, (through regulation of the IL-4R) or IL-17A to influence cell infiltration and tissue remodeling. OSM reduces concentrations of IL-4, IL-13, and IL-17A required to elicit robust local inflammatory and lung remodeling responses.

Increases in ECM are a central effect of diseases generating pulmonary fibrosis including conditions upon exposure to asbestos or silica, or that of unknown origin in IPF. Connective tissue cells are important targets of various molecules that induce collagens and other ECM components that contribute to the pathologic accumulation of matrix. The development of ECM remodeling in chronic lung inflammation could be dependent, at least in part, on OSM and/or its combinatorial effects with other cytokines on a variety of cells that can contribute to the inflammation and ECM deposits, including the lung fibroblast, lung myofibroblast, smooth muscle cells, and epithelial cells (see Figure 4).

### 10.1. Inflammation

Studies of gp130 cytokine overexpression *in vivo* were completed by DiCosmo et al. and his group, where transgenic mice overexpressing IL-6 in airway epithelial cells [215] showed increased CD4+ T cells and B220+ cell infiltration into lung and decreased sensitivity to methacholine in airway hyperreactivity assays. Their further studies showed that transgenic IL-11 epithelial overexpression resulted in increased airway subepithelial fibrosis compared to IL-6 transgene [216, 217]. In using the adenovirus-mediated transient pulmonary transgenic overexpression system, it was later shown that mouse OSM induced a marked increase in airway ECM accumulation as well as increases in eosinophil infiltration in C57Bl/6 mice [218], whereas overexpression of IL-6 using the same adenovirus vector system in the rat had previously been shown to mediate increases in lymphocyte accumulation and antibody levels but did not result in the changes in ECM deposition [219, 220]. These data suggest that OSM recruits additional pathways *in vivo* in the rodent lung further than those of IL-6 or IL-11. Recently we have shown that the AdOSM system induces inflammatory chemokines and cell infiltration that is largely dependent on IL-6; however, induction of accumulation/activation of B cells, dendritic cells, and CD4T cells did not require IL-6 [79], nor did the formation of induced bronchus associated lymphoid tissue (iBalt). This data implicates activities of OSM in lung *in vivo* that do not overlap with IL-6.

In contrast to effects in joints, overexpression of mouse OSM in lungs via Adenovirus vector (AdmOSM) induces marked eosinophil infiltration accompanied by elevation in eosinophil chemotactic factors [218, 221] and is characterized by increased collagen, fibronectin, and *α*-smooth muscle actin (SMA). In the mouse system, OSM induced eotaxin-1 in fibroblasts [218]* in vitro*, as well as expression of VCAM-1 by fibroblasts [63] which likely contribute to the attraction and passage of eosinophils through lung tissue compartments. The inflammatory effects of OSM overexpression are thus dependent on the site of transient overexpression, and the net result is likely due to differing cytokine milieu that is also present in a tissue-specific manner.

### 10.2. Airway Smooth Muscle Cells

The identification of OSM and its ability to induce eosinophil chemotactic factor expression in the mouse system [218] was complimented by studies investigating the effects of OSM on other lung cell populations. S. Shore's group generated a series of studies using human airway smooth muscle cell cultures and showed that human OSM and IL-6 (but not IL-11) induced STAT-3 activation and could synergize with IL-1 in upregulating COX-2 and PGE2 release [222]; the eotaxin-1 expression induced by IL-4 or IL-13 [223] could be synergistically enhanced with costimulation with OSM but not other gp130 cytokines [224], and this was associated with the induction of the receptor chain IL-4R*α*; human OSM induced VEGF expression and also synergized with IL-1*β* in the regulation of VEGF [225], and this was associated with the upregulation of the IL-1Receptor.

### 10.3. Lung Fibroblasts

Effects of OSM on human lung fibroblasts in culture have been identified including older studies showing upregulation of TIMP-1 and synergistic upregulation of IL-6 when OSM was combined with IL-1*α* to stimulate cells [226]. Human lung fibroblast proliferation and collagen synthesis (but not apoptosis) can be induced by OSM [227]. These effects could be inhibited by pharmacological agents that inhibited MAPkinases or tyrosine kinases and also suggest a significant role in lung inflammatory diseases [228]. Hepatocyte growth factor expression could also be elevated by OSM in lung fibroblast cell cultures [229]. The studies showing synergistic actions of OSM with IL-4 or with IL-13 in the induction of chemotactic factors such as eotaxin-1 were reproduced using mouse lung fibroblasts in a STAT-6 dependent mechanism [63] and were also associated with elevation of IL-4R*α* [230]. These data point to the potential for OSM to combine with Th2 cytokines in mediating lung pathology and the generation of both inflammatory cell accumulation as well as ECM deposition. Cell signaling in fibroblasts in response to OSM includes the activation of STAT-1, -3, -5, -6, as well as p38 MAPK, ERK-1, -2, and protein Kinase C delta [62–64, 231] *in vitro*, indicating that a variety of fibroblasts functions can be modified by OSM.

Although TGF*β*-SMAD3 signaling has been considered a central mediator in the generation of fibrotic responses and *α*-SMA expressing myofibroblasts, very recent data using human lung fibroblast cultures have shown that OSM can enhance gel contraction and *α*-SMA expression *in vitro* through a STAT3-mediated pathway that is independent *in vitro* on TGF*β* or PGE [232]. The ability of OSM to induce a myofibroblast-like phenotype in these cells suggests the contribution of this pathway to myofibroblast accumulation in lung conditions involving fibrosis. STAT3 activation may be central to mechanism in conditions such as pulmonary fibrosis [233]. Hyperelevated activation of STAT-3 in a genetic mouse model system has also recently been shown to render animals highly susceptible to fibrosis in the bleomycin model of lung fibrosis, whereas hypoactivation of STAT3 rendered these mice protected [234]. Induction of ECM by overexpression byAdOSM in mouse lung did not absolutely require TGF*β*-SMAD3 signaling and indeed induces sustained STAT3 activation [234]. While many different cytokines can activate STAT3, sustained activation of STAT3 by OSM may be indicative of OSM/STAT3 signaling as prominent in ECM remodeling in lung in certain conditions.

### 10.4. Epithelial Cells and EMT

Damage to epithelium and activation of epithelial wound healing mechanisms are thought to play important roles in fibrosis of tissues at mucosal surfaces [235]. Lung epithelial cells of various derivations respond to OSM *in vitro*, including the adenocarcinoma A549 type 2 alveolar line with induction of *α*-1 antitrypsin [236] and increases in alkaline phosphatase [237]. OSM synergized with house dust mite extract to induce PGE2 in these cells *in vitro* [238]. Distinct signaling pathways in these cells by OSM compared to IL-31 or other gp130 cytokines have also been noted [66]. Secretoglobins can be regulated by OSM in transformed Clara cells [239]. Cell cultures from normal human bronchial brushings respond robustly to OSM, moderately to IL-6, and little to LIF *in vitro* [182]. Lung epithelial cells also respond to OSM and IL-4 with synergy in expression of chemokines such as eotaxin-1 (C. D. Richards, unpublished). Although OSM has been shown to induce EMT in a transformed lung epithelial cell line [183], it is not clear if nontransformed lung epithelial cells behave similarly.

Interestingly, kidney proximal tubule cells were shown to undergo EMT in response to peripheral blood mononuclear-conditioned medium and to OSM *in vitro* [240]. On the other hand, Sarközi et al. [241] completed work showing that OSM inhibited TGF*β*-induced EMT in human proximal tubule cells. Tissues from patients with chronic obstructive nephropathy displayed elevated OSM expression, and in the mouse model of unilateral ureteral obstruction (UUO), kidney expression of OSM and the OSMR*β* subunit were elevated within a short period of time following obstruction [242]. Studies have also shown suppression of renin in a kidney cell line by OSM that requires STAT5 [243]. These data suggest that further studies clarifying functions of OSM in kidney disease are merited.

## 11. OSM and Inflammatory Skin Conditions

Studies examining functions of OSM on cells derived from skin date back to the work of Duncan et al. [244] who showed *in vitro* that human OSM could regulate collagen and glycosaminoglycan production, as well as mRNA transcripts for collagen I and III, hyaluronic acid, chondroitin, and dermatan sulphate in microcultures of skin fibroblasts. In examining the molecular mechanisms of collagen gene induction, Ihn et al. [245] showed that Sp1/Sp3 transcription factors bind to regions in the collagen *α*-2 (I) promoter and were important in OSM responsiveness, although changes to the binding of this particular site were not readily apparent due to OSM stimulation. Later studies showed that OSM induced dermal fibroblast growth through MAPKinase signalling [246] and could potently regulate chemokine expression in these cells [247]. Peripheral blood monocytes from patients with systemic sclerosis produced more OSM than those from control patients [248] indicating potential participation in manifestation of skin disorders.

Transcriptional studies on keratinocytes indicated that OSM could regulate many genes including those of growth factors, angiogenesis, and tissue remodelling [249]. OSM induced keratinocyte migration and reconstituted epidermal thickness [250]. Moreover, these investigators showed that the receptor chains gp130 and OSMR*β*, but not LIFR chain, were present in keratinocytes indicating functional type II OSM receptors and that both OSM and OSMR*β* transcripts were elevated in skin lesions from both psoriatic patients and atopic dermatitis [250] compared to healthy skin samples. This study also showed that OSM was expressed in lymphocyte populations derived from both psoriasis and dermatitis lesions. OSM appears to act in concert with other skin inflammatory mediators IL-1*α*, TNF, IL-17A, and IL-22 to regulate several genes implicated in psoriasis, including S100A9 and several chemokines [251]. Giot et al. [252] have recently shown that OSM is expressed in hypertensive leg ulcer lesions and contributes along with IL-1*β* to the maximizing of lesion pathology. Thus OSM interacts in networks of cytokines in skin disease, and how IL-31 fits into this network as a mediator in pruritic skin conditions [253] will be intriguing.

## 12. Atherosclerosis and Cardiovascular Disease

Atherosclerosis is currently widely accepted as an inflammatory condition involving aspects of both the innate and the acquired arms of the immune system. The atherosclerotic lesion is characterized by endothelial cell activation resulting from damage due to a variety of stimuli and this leads to accumulation of infiltrating inflammatory cells, particularly macrophages and lymphocytes. Cells such as macrophages acquire large amounts of cholesterol and become foam cells, and over time atherosclerotic plaques undergo substantial changes with continued cell infiltration, the formation of necrotic cores, proliferation and migration of smooth muscle cells, and extracellular matrix remodelling. The role of inflammation in atherosclerosis has been increasing in prominence as a major factor in etiopathogenesis (reviewed in [254–256]); however, the precise mechanisms of inflammation in vessel walls in atherosclerotic disease are not definitively characterized. Pro-inflammatory or innate cytokines released by macrophages including IL-1 and TNF have been implicated in the development of atherosclerosis in mouse models [257] whereas opposing roles of IL-6 have been described [258, 259] that may be dependent on the experimental model used.

The involvement of macrophages is clearly indicated in development of early plaque as well as abundance in ruptured plaques [260, 261]. Macrophages are primary sources of OSM and IL-6, and the effects of OSM in atherosclerosis and cardiovascular disease is becoming apparent. Several studies have examined OSM stimulation of endothelial cells, including expression of IL-6 [262], G-CSF, and GM-CSF [263], adhesion molecules such as p-selectin, l-selectin, and VCAM-1 *in vitro* [264, 265], and increased neutrophil rolling and adhesion to human umbilical cord endothelium in flow conditions [266]. OSM also induced microvascular endothelial cell angiogenesis [267] associated with COX-2 elevation, elevated Angiopoietin-2 [268], and showed angiogenic activity *in vivo* in rabbits [269]. Furthermore, OSM action has been observed in vascular smooth muscle cells (SMC) including proliferation of rabbit aortic SMC [270] and induction of VEGF, IL-6, and COX-2 in human vascular SMC [271, 272]. Collectively, the data suggest a proatherosclerotic function of OSM.

OSM ligand could be detected in human aortic aneurism tissue [264], and more recently OSM ligand was detected in both human aortic atherosclerotic lesions and in ApoE knockout mouse atherosclerotic lesions [273]. OSM functions in atherosclerosis are likely complex on the basis of its ability to regulate endothelial cells, vascular SMC, and angiogenesis. Likewise, the functions of OSM in myocardial infarction and remodelling are complex. The identification of regulation by OSM of cardiomyocyte expression of PAI-1 [274], VEGF [275], and TIMP-1 [276] and later SDF-1 [277] suggested roles in inflammation, repair, and tissue regeneration of damaged heart tissue. Kubin et al. [278] completed work to show that OSM mediates cardiac myocyte dedifferentiation *in vitro* and *in vivo*, can upregulate stem cell markers, and improve cardiac function after infarct but also has a deleterious effect in models of chronic dilated cardiomyopathy. Further study would help clarify potential for modification of OSM or its signalling for a therapeutic goal in cardiac and/or vascular disease.

## 13. Summary

Accumulated literature has described an array of OSM homeostatic functions including hematopoiesis, bone and fat turnover, CNS development, and liver regeneration. Data showing pro-inflammatory roles of OSM in joint, skin, lung, and vascular diseases are compelling and collectively suggest that OSM actions *in vivo* depend on context of the particular tissue/organ and cytokine milieu present. Thus OSM amplifies inflammatory responses differently in specific tissues, reduces thresholds of cytokine needed to initiate tissue inflammation and pathology, and likely does so in part through upregulation of receptors for other cytokines. Its role in cancers depends on the cancer type, where along with antiproliferative actions it also imparts an increased invasive phenotype in some tumours, whereas in others OSM can stimulate proliferation of the cancer cells. With effects in inflammation and angiogenesis, OSM may also play a role in modulating tumour microenvironment that facilitates tumour growth.

OSM has unique functions from other gp130 cytokines both *in vitro* and *in vivo *that involve pathways specifically invoked by the OSMR*β*/gp130 or type II OSM receptor. However, since its activities range over so many cell types, targeting of either reduced- or enhanced-OSM signaling to specific tissues or organs, in a time sensitive fashion, would be required to enable approaches with less undesirable or unpredicted side effects. Generating phenotypic changes *ex vivo* in precursor cells such as MSC and reintroducing these for benefit, for example, in bone repair, may be possible in the future. The differences in species specificity of the ligand and receptor interactions complicate the development of preclinical models in rodents, although use of rat models may assist with this issue. In summary, OSM has shown to be an intriguing cytokine with still more to understand about its biology, due to its pleiotropic nature and ability to regulate several organ systems, if one were to effectively manipulate its function for therapeutic benefit.

## References

[1] Tanaka M, Miyajima A (2003). Oncostatin M, a multifunctional cytokine. *Reviews of Physiology, Biochemistry and Pharmacology*.

[2] Chen S-H, Benveniste EN (2004). Oncostatin M: a pleiotropic cytokine in the central nervous system. *Cytokine and Growth Factor Reviews*.

[3] Silver JS, Hunter CA (2010). gp130 at the nexus of inflammation, autoimmunity, and cancer. *Journal of Leukocyte Biology*.

[4] Sims NA, Walsh NC (2010). GP130 cytokines and bone remodelling in health and disease. *BMB Reports*.

[5] Zarling JM, Shoyab M, Marquardt H (1986). Oncostatin M: a growth regulator produced by differentiated histiocytic lymphoma cells. *Proceedings of the National Academy of Sciences of the United States of America*.

[6] Malik N, Kallestad JC, Gunderson NL (1989). Molecular cloning, sequence analysis, and functional expression of a novel growth regulator, oncostatin M. *Molecular and Cellular Biology*.

[7] Linsley PS, Kallestad J, Ochs V, Neubauer M (1990). Cleavage of a hydrophilic C-terminal domain increases growth-inhibitory activity of oncostatin M. *Molecular and Cellular Biology*.

[8] Yoshimura A, Ichihara M, Kinjyo I (1996). Mouse oncostatin M: an immediate early gene induced by multiple cytokines through the JAK-STAT5 pathway. *EMBO Journal*.

[9] Okaya A, Kitanaka J, Kitanaka N (2005). Oncostatin M inhibits proliferation of rat oval cells, OC15-5, inducing differentiation into hepatocytes. *American Journal of Pathology*.

[10] Bravo J, Heath JK (2000). Receptor recognition by gp130 cytokines. *The EMBO Journal*.

[11] Deller MC, Hudson KR, Ikemizu S, Bravo J, Jones EY, Heath JK (2000). Crystal structure and functional dissection of the cytostatic cytokine oncostatin M. *Structure*.

[12] Plun-Favreau H, Perret D, Diveu C (2003). Leukemia inhibitory factor (LIF), cardiotrophin-1, and oncostatin M share structural binding determinants in the immunoglobulin-like domain of LIF receptor. *The Journal of Biological Chemistry*.

[13] Somasundaram R, Ruehl M, Schaefer B (2002). Interstitial collagens I, III, and VI sequester and modulate the multifunctional cytokine oncostatin M. *The Journal of Biological Chemistry*.

[14] Suda T, Chida K, Todate A (2002). Oncostatin M production by human dendritic cells in response to bacterial products. *Cytokine*.

[15] Grenier A, Dehoux M, Boutten A (1999). Oncostatin M production and regulation by human polymorphonuclear neutrophils. *Blood*.

[16] Grenier A, Combaux D, Chastre J (2001). Oncostatin M production by blood and alveolar neutrophils during acute lung injury. *Laboratory Investigation*.

[17] Guillet C, Fourcin M, Chevalier S, Pouplard A, Gascan H (1995). ELISA detection of circulating levels of LIF, OSM, and CNTF in septic shock. *Annals of the New York Academy of Sciences*.

[18] Hurst SM, McLoughlin RM, Monslow J (2002). Secretion of oncostatin M by infiltrating neutrophils: regulation of IL-6 and chemokine expression in human mesothelial cells. *Journal of Immunology*.

[19] Ganesh K, Das A, Dickerson R (2012). Prostaglandin E_2_ induces oncostatin M expression in human chronic wound macrophages through Axl receptor tyrosine kinase pathway. *The Journal of Immunology*.

[20] Tamura S, Morikawa Y, Miyajima A, Senba E (2002). Expression of oncostatin M in hematopoietic organs. *Developmental Dynamics*.

[21] Salamon P, Shoham NG, Puxeddu I, Paitan Y, Levi-Schaffer F, Mekori YA (2008). Human mast cells release oncostatin M on contact with activated T cells: possible biologic relevance. *Journal of Allergy and Clinical Immunology*.

[22] Hilton DJ (1992). LIF: lots of interesting functions. *Trends in Biochemical Sciences*.

[23] Kishimoto T, Akira S, Narazaki M, Taga T (1995). Interleukin-6 family of cytokines and gp130. *Blood*.

[24] Kamimura D, Ishihara K, Hirano T (2003). IL-6 signal transduction and its physiological roles: the signal orchestration model. *Reviews of physiology, biochemistry and pharmacology*.

[25] Murakami M, Kamimura D, Hirano T (2004). New IL-6 (gp130) family cytokine members, CLC/NNT1/BSF3 and IL-27. *Growth Factors*.

[26] Villarino AV, Huang E, Hunter CA (2004). Understanding the pro- and anti-inflammatory properties of IL-27. *The Journal of Immunology*.

[27] Senaldi G, Varnum BC, Ulla Sarmiento CS (1999). Novel neurotrophin-1/B cell-stimulating factor-3: a cytokine of the IL-6 family. *Proceedings of the National Academy of Sciences of the United States of America*.

[28] Artis D, Villarino A, Silverman M (2004). The IL-27 receptor (WSX-1) is an inhibitor of innate and adaptive elements of type 2 immunity. *The Journal of Immunology*.

[29] Taga T (1997). The signal transducer gp130 is shared by interleukin-6 family of haematopoietic and neurotrophic cytokines. *Annals of Medicine*.

[30] Heinrich PC, Behrmann I, Müller-Newen G, Schaper F, Graeve L (1998). Interleukin-6-type cytokine signalling through the gp130/Jak/STAT pathway. *Biochemical Journal*.

[31] Behrmann I, Hermanns HM, Haan C (2000). Signalling of interleukin-6 type cytokines via gp130, leukemia inhibitory factor (LIF) receptor and oncostatin M receptor. *European Cytokine Network*.

[32] Dillon SR, Sprecher C, Hammond A (2004). Interleukin 31, a cytokine produced by activated T cells, induces dermatitis in mice. *Nature Immunology*.

[33] Taga T (1997). gp130 and the interleukin-6 family of cytokines. *Annual Review of Immunology*.

[34] Heinrich PC, Behrmann I, Haan S, Hermanns HM, Müller-Newen G, Schaper F (2003). Principles of interleukin (IL)-6-type cytokine signalling and its regulation. *Biochemical Journal*.

[35] Chevalier S, Fourcin M, Robledo O, Wijdenes J, Pouplard-Barthelaix A, Gascan H (1996). Interleukin-6 family of cytokines induced activation of different functional sites expressed by gp130 transducing protein. *The Journal of Biological Chemistry*.

[36] Liu J, Modrell B, Aruffo A, Scharnowske S, Shoyab M (1994). Interactions between oncostatin M and the IL-6 signal transducer, gp130. *Cytokine*.

[37] Gearing DP, Comeau MR, Friend DJ (1992). The IL-6 signal transducer, gp130: an oncostatin M receptor and affinity converter for the LIF receptor. *Science*.

[38] Mosley B, De Imus C, Friend D (1996). Dual oncostatin M (OSM) receptors. Cloning and characterization of an alternative signaling subunit conferring OSM-specific receptor activation. *The Journal of Biological Chemistry*.

[39] Fritz DK (2010). The role of the oncostatin M—STAT6 axis in pulmonary eosinophilia, goblet cell hyperplasia, airway hyperresponsiveness and pulmonary fibrosis. *ETD Collection For McMaster University*.

[40] Ichihara M, Hara T, Kim H, Murate T, Miyajima A (1997). Oncostatin M and leukemia inhibitory factor do not use the same functional receptor in mice. *Blood*.

[41] Lindberg RA, Juan TS-C, Welcher AA (1998). Cloning and characterization of a specific receptor for mouse oncostatin M. *Molecular and Cellular Biology*.

[42] Tanaka M, Hara T, Copeland NG, Gilbert DJ, Jenkins NA, Miyajima A (1999). Reconstitution of the functional mouse oncostatin M (OSM) receptor: molecular cloning of the mouse OSM receptor *β* subunit. *Blood*.

[43] Drechsler J, Grotzinger J, Hermanns HM (2012). Characterization of the rat oncostatin M receptor complex which resembles the human, but differs from the murine cytokine receptor. *PLoS ONE*.

[44] Soldi R, Graziani A, Benelli R (1994). Oncostatin M activates phosphatidylinositol-3-kinase in Kaposi’s sarcoma cells. *Oncogene*.

[45] Faris M, Ensoli B, Stahl N (1996). Differential activation of the extracellular signal-regulated kinase, Jun Kinase and Janus Kinase-Stat pathways by oncostatin M and basic fibroblast growth factor in AIDS-derived Kaposi’s sarcoma cells. *AIDS*.

[46] Ernst M, Oates A, Dunn AR (1996). gp130-mediated signal transduction in embryonic stem cells involves activation of Jak and ras/mitogen-activated protein kinase pathways. *The Journal of Biological Chemistry*.

[47] Symes A, Gearan T, Eby J, Fink JS (1997). Integration of Jak-Stat and AP-1 signaling pathways at the vasoactive intestinal peptide cytokine response element regulates ciliary neurotrophic factor-dependent transcription. *The Journal of Biological Chemistry*.

[48] Stancato LF, Sakatsume M, David M (1997). Beta interferon and oncostatin M activate Raf-1 and mitogen-activated protein kinase through a JAK1-dependent pathway. *Molecular and Cellular Biology*.

[49] Pfitzner E, Kliem S, Baus D, Litterst CM (2004). The role of STATs in inflammation and inflammatory diseases. *Current Pharmaceutical Design*.

[50] Horvath CM (2000). STAT proteins and transcriptional responses to extracellular signals. *Trends in Biochemical Sciences*.

[51] Takeda K, Akira S (2000). STAT family of transcription factors in cytokine-mediated biological responses. *Cytokine and Growth Factor Reviews*.

[52] Hendry L, John S (2004). Regulation of STAT signalling by proteolytic processing. *European Journal of Biochemistry*.

[53] Chen XP, Losman JA, Rothman P (2000). SOCS proteins, regulators of intracellular signaling. *Immunity*.

[54] Liu B, Liao J, Rao X (1998). Inhibition of Stat1-mediated gene activation by PIAS1. *Proceedings of the National Academy of Sciences of the United States of America*.

[55] Tang D, Okada H, Ruland J (2001). Akt is activated in response to an apoptotic signal. *The Journal of Biological Chemistry*.

[56] Kennedy SG, Wagner AJ, Conzen SD (1997). The PI 3-kinase/Akt signaling pathway delivers an anti-apoptotic signal. *Genes and Development*.

[57] Zhang HG, Wang Y, Xie JF (2001). Regulation of tumor necrosis factor alpha-mediated apoptosis of rheumatoid arthritis synovial fibroblasts by the protein kinase Akt. *Arthritis & Rheumatism*.

[58] Chang L, Karin M (2001). Mammalian MAP kinase signalling cascades. *Nature*.

[59] Auguste P, Guillet C, Fourcin M (1997). Signaling of type II oncostatin M receptor. *The Journal of Biological Chemistry*.

[60] Hermanns HM, Radtke S, Schaper F, Heinrich PC, Behrmann I (2000). Non-redundant signal transduction of interleukin-6-type cytokines: the adapter protein Shc is specifically recruited to the oncostatin M receptor. *The Journal of Biological Chemistry*.

[61] Stross C, Radtke S, Clahsen T (2006). Oncostatin M receptor-mediated signal transduction is negatively regulated by SOCS_3_ through a receptor tyrosine-independent mechanism. *The Journal of Biological Chemistry*.

[62] Tong L, Smyth D, Kerr C, Catterall J, Richards CD (2004). Mitogen-activated protein kinases Erk1/2 and p38 are required for maximal regulation of TIMP-1 by oncostatin M in murine fibroblasts. *Cellular Signalling*.

[63] Fritz DK, Kerr C, Tong L, Smyth D, Richards CD (2006). Oncostatin-M up-regulates VCAM-1 and synergizes with IL-4 in eotaxin expression: involvement of STAT6. *The Journal of Immunology*.

[64] Smyth DC, Kerr C, Richards CD (2006). Oncostatin M-induced IL-6 expression in murine fibroblasts requires the activation of protein kinase C*δ*. *The Journal of Immunology*.

[65] Diveu C, Lak-Hal A-HL, Froger J (2004). Predominant expression of the long isoform of GP130-like (GPL) receptor is required for interleukin-31 signaling. *European Cytokine Network*.

[66] Chattopadhyay S, Tracy E, Liang P, Robledo O, Rose-John S, Baumann H (2007). Interleukin-31 and oncostatin-M mediate distinct signaling reactions and response patterns in lung epithelial cells. *The Journal of Biological Chemistry*.

[67] Jawa RS, Chattopadhyay S, Tracy E (2008). Regulated expression of the IL-31 receptor in bronchial and alveolar epithelial cells, pulmonary fibroblasts, and pulmonary macrophages. *Journal of Interferon and Cytokine Research*.

[68] Bilsborough J, Leung DYM, Maurer M (2006). IL-31 is associated with cutaneous lymphocyte antigen-positive skin homing T cells in patients with atopic dermatitis. *Journal of Allergy and Clinical Immunology*.

[69] Nobbe S, Dziunycz P, Mühleisen B (2012). IL-31 expression by inflammatory cells is preferentially elevated in atopic dermatitis. *Acta Dermato-Venereologica*.

[70] Perrigoue JG, Li J, Zaph C (2007). IL-31-IL-31R interactions negatively regulate type 2 inflammation in the lung. *Journal of Experimental Medicine*.

[71] Bilsborough J, Mudri S, Chadwick E, Harder B, Dillon SR (2010). IL-31 receptor (IL-31RA) knockout mice exhibit elevated responsiveness to oncostatin M. *The Journal of Immunology*.

[72] Diveu C, Venereau E, Froger J (2006). Molecular and functional characterization of a soluble form of oncostatin M/interleukin-31 shared receptor. *The Journal of Biological Chemistry*.

[73] Jostock T, Müllberg J, Özbek S (2001). Soluble gp130 is the natural inhibitor of soluble interleukin-6 receptor transsignaling responses. *European Journal of Biochemistry*.

[74] Rose-John S, Heinrich PC (1994). Soluble receptors for cytokines and growth factors: generation and biological function. *Biochemical Journal*.

[75] Brolund L, Küster A, Korr S, Vogt M, Müller-Newen G (2011). A receptor fusion protein for the inhibition of murine oncostatin M. *BMC Biotechnology*.

[76] Malik N, Haugen HS, Modrell B, Shoyab M, Clegg CH (1995). Developmental abnormalities in mice transgenic for bovine oncostatin M. *Molecular and Cellular Biology*.

[77] Bamber B, Reife RA, Haugen HS, Clegg CH (1998). Oncostatin M stimulates excessive extracellular matrix accumulation in a transgenic mouse model of connective tissue disease. *Journal of Molecular Medicine*.

[78] Clegg CH, Rulffes JT, Wallace PM, Haugen HS (1996). Regulation of an extrathymic T-cell development pathway by oncostatin M. *Nature*.

[79] Botelho FM, Rangel-Moreno J, Fritz D, Randall TD, Xing Z, Richards CD (2013). Pulmonary expression of oncostatin M, (OSM) promotes inducible BALT formation independently of IL-6, despite a role for IL-6 in OSM-driven pulmonary inflammation. *The Journal of Immunology*.

[80] Morikawa Y, Tamura S, Minehata K-I, Donovan PJ, Miyajima A, Senba E (2004). Essential function of oncostatin M in nociceptive neurons of dorsal root ganglia. *Journal of Neuroscience*.

[81] Esashi E, Ito H, Minehata K-I, Saito S, Morikawa Y, Miyajima A (2009). Oncostatin M deficiency leads to thymic hypoplasia, accumulation of apoptotic thymocytes and glomerulonephritis. *European Journal of Immunology*.

[82] Clegg CH, Haugen HS, Rulffes JT, Friend SL, Farr AG (1999). Oncostatin M transforms lymphoid tissue function in transgenic mice by stimulating lymph node T-cell development and thymus autoantibody production. *Experimental Hematology*.

[83] Tanaka M, Hirabayashi Y, Sekiguchi T, Inoue T, Katsuki M, Miyajima A (2003). Targeted disruption of oncostatin M receptor results in altered hematopoiesis. *Blood*.

[84] Hams E, Colmont CS, Dioszeghy V (2008). Oncostatin M receptor-*β* signaling limits monocytic cell recruitment in acute inflammation. *The Journal of Immunology*.

[85] Nakamura K, Nonaka H, Saito H, Tanaka M, Miyajima A (2004). Hepatocyte proliferation and tissue remodeling is impaired after liver injury in oncostatin M receptor knockout mice. *Hepatology*.

[86] Walker EC, McGregor NE, Poulton IJ (2010). Oncostatin M promotes bone formation independently of resorption when signaling through leukemia inhibitory factor receptor in mice mice. *The Journal of Clinical Investigation*.

[87] Komori T, Tanaka M, Senba E, Miyajima A, Morikawa Y (2013). Lack of oncostatin M receptor beta Leads to adipose tissue inflammation and insulin resistance by switching macrophage phenotype. *The Journal of Biological Chemistry*.

[88] Baumann H, Gauldie J (1994). The acute phase response. *Immunology Today*.

[89] Richards CD, Brown TJ, Shoyab M, Baumann H, Gauldie J (1992). Recombinant oncostatin M stimulates the production of acute phase proteins in HepG2 cells and rat primary hepatocytes in vitro. *The Journal of Immunology*.

[90] Grove RI, Mazzucco CE, Radka SF, Shoyab M, Kiener PA (1991). Oncostatin M up-regulates low density lipoprotein receptors in HepG2 cells by a novel mechanism. *The Journal of Biological Chemistry*.

[91] Kanda J, Uchiyama T, Tomosugi N, Higuchi M, Uchiyama T, Kawabata H (2009). Oncostatin M and leukemia inhibitory factor increase hepcidin expression in hepatoma cell lines. *International Journal of Hematology*.

[92] Chung B, Verdier F, Matak P, Deschemin J-C, Mayeux P, Vaulont S (2010). Oncostatin M is a potent inducer of hepcidin, the iron regulatory hormone. *FASEB Journal*.

[93] Miyajima A, Kinoshita T, Tanaka M, Kamiya A, Mukouyama Y, Hara T (2000). Role of oncostatin M in hematopoiesis and liver development. *Cytokine and Growth Factor Reviews*.

[94] Hamada T, Sato A, Hirano T (2007). Oncostatin M gene therapy attenuates liver damage induced by dimethylnitrosamine in rats. *American Journal of Pathology*.

[95] Mann DA, Marra F (2010). Fibrogenic signalling in hepatic stellate cells. *Journal of Hepatology*.

[96] Znoyko I, Sohara N, Spicer SS, Trojanowska M, Reuben A (2005). Expression of oncostatin M and its receptors in normal and cirrhotic human liver. *Journal of Hepatology*.

[97] Tanaka T, Narazaki M, Kishimoto T (2011). Anti-interleukin-6 receptor antibody, tocilizumab, for the treatment of autoimmune diseases. *FEBS Letters*.

[98] Cawston TE, Curry VA, Summers CA (1998). The role of oncostatin M in animal and human connective tissue collagen turnover and its localization within the rheumatoid joint. *Arthritis & Rheumatism*.

[99] Langdon C, Leith J, Smith F, Richards CD (1997). Oncostatin M stimulates monocyte chemoattractant protein-1- and interleukin-1-induced matrix metalloproteinase-1 production by human synovial fibroblasts in vitro. *Arthritis and Rheumatism*.

[100] Hui W, Bell M, Carroll G (1997). Detection of oncostatin M in synovial fluid from patients with rheumatoid arthritis. *Annals of the Rheumatic Diseases*.

[101] Richards CD, Shoyab M, Brown TJ, Gauldie J (1993). Selective regulation of metalloproteinase inhibitor (TIMP-1) by oncostatin M in fibroblasts in culture. *The Journal of Immunology*.

[102] Li WQ, Zafarullah M (1998). Oncostatin M up-regulates tissue inhibitor of metalloproteinases-3 gene expression in articular chondrocytes viade novo transcription, protein synthesis, and tyrosine kinase- and mitogen-activated protein kinase- dependent mechanisms. *The Journal of Immunology*.

[103] Langdon C, Kerr C, Hassen M, Hara T, Arsenault AL, Richards CD (2000). Murine oncostatin M stimulates mouse synovial fibroblasts in vitro and induces inflammation and destruction in mouse joints in vivo. *American Journal of Pathology*.

[104] Hintzen C, Quaiser S, Pap T, Heinrich PC, Hermanns HM (2009). Induction of CCL13 expression in synovial fibroblasts highlights a significant role of oncostatin M in rheumatoid arthritis. *Arthritis and Rheumatism*.

[105] Cawston T, Billington C, Cleaver C (1999). The regulation of MMPs and TIMPs in cartilage turnover. *Annals of the New York Academy of Sciences*.

[106] Rowan AD, Koshy PJ, Shingleton WD (2001). Synergistic effects of glycoprotein 130 binding cytokines in combination with interleukin-1 on cartilage collagen breakdown. *Arthritis & Rheumatism*.

[107] Koshy PJT, Lundy CJ, Rowan AD (2002). The modulation of matrix metalloproteinase and ADAM gene expression in human chondrocytes by interleukin-1 and oncostatin M: a time-course study using real-time quantitative reverse transcription-polymerase chain reaction. *Arthritis and Rheumatism*.

[108] Barksby HE, Hui W, Wappler I (2006). Interleukin-1 in combination with oncostatin M up-regulates multiple genes in chondrocytes: implications for cartilage destruction and repair. *Arthritis and Rheumatism*.

[109] Koshy PJ, Henderson N, Logan C, Life PF, Cawston TE, Rowan AD (2002). Interleukin 17 induces cartilage collagen breakdown: novel synergistic effects in combination with proinflammatory cytokines. *Annals of the Rheumatic Diseases*.

[110] Plater-Zyberk C, Buckton J, Thompson S (2001). Amelioration of arthritis in two murine models using antibodies to oncostatin M. *Arthritis Rheum*.

[111] Wallace PM, MacMaster JF, Rouleau KA (2000). Erratum: Regulation of inflammatory responses by oncostatin M. *The Journal of Immunology*.

[112] Wallace PM, MacMaster JF, Rouleau KA (2000). Erratum: regulation of inflammatory responses by oncostatin M. *The Journal of Immunology*.

[113] Clegg CH, Haugen HS, Rulffes JT, Friend SL, Farr AG (1999). Oncostatin M transforms lymphoid tissue function in transgenic mice by stimulating lymph node T-cell development and thymus autoantibody production. *Experimental Hematology*.

[114] Juan TS-C, Bolon B, Lindberg RA, Sun Y, Van G VG, Fletcher FA (2009). Mice overexpressing murine oncostatin m (osm) exhibit changes in hematopoietic and other organs that are distinct from those of mice overexpressing human OSM or bovine OSM. *Veterinary Pathology*.

[115] Lubberts E, Joosten LAB, Chabaud M (2000). IL-4 gene therapy for collagen arthritis suppresses synovial IL-17 and osteoprotegerin ligand and prevents bone erosion. *The Journal of Clinical Investigation*.

[116] Lubberts E, Joosten LAB, Van Den Bersselaar L (2000). Intra-articular IL-10 gene transfer regulates the expression of collagen-induced arthritis (CIA) in the knee and ipsilateral paw. *Clinical and Experimental Immunology*.

[117] De Hooge ASK, Van de Loo FAJ, Bennink MB (2003). Growth plate damage, a feature of juvenile idiopathic arthritis, can be induced by adenoviral gene transfer of oncostatin M: a comparative study in gene-deficient mice. *Arthritis and Rheumatism*.

[118] Rowan A, Hui W, Cawston TE, Richards CD (2003). Adenoviral gene transfer of interleukin-1 in combination with oncostatin M induces significant joint damage in a murine model. *American Journal of Pathology*.

[119] Hui W, Cawston TE, Richards CD, Rowan AD (2005). A model of inflammatory arthritis highlights a role for oncostatin M in pro-inflammatory cytokine-induced bone destruction via RANK/RANKL. *Arthritis Research & Therapy*.

[120] Hui W, Rowan AD, Richards CD, Cawston TE (2003). Oncostatin M in combination with tumor necrosis factor *α* induces cartilage damage and matrix metalloproteinase expression in vitro and in vivo. *Arthritis and Rheumatism*.

[121] Barksby HE, Milner JM, Patterson AM (2006). Matrix metalloproteinase 10 promotion of collagenolysis via procollagenase activation: implications for cartilage degradation in arthritis. *Arthritis and Rheumatism*.

[122] Milner JM, Kevorkian L, Young DA (2006). Fibroblast activation protein alpha is expressed by chondrocytes following a pro-inflammatory stimulus and is elevated in osteoarthritis. *Arthritis Research and Therapy*.

[123] Litherland GJ, Dixon C, Lakey RL (2008). Synergistic collagenase expression and cartilage collagenolysis are phosphatidylinositol 3-kinase/Akt signaling-dependent. *The Journal of Biological Chemistry*.

[124] Gilbert SJ, Blain EJ, Al-Sabah A, Zhang Y, Duance VC, Mason DJ (2012). Protein kinase R plays a pivotal role in oncostatin M and interleukin-1 signalling in bovine articular cartilage chondrocytes. *European Cells and Materials*.

[125] Tanaka T, Narazaki M, Kishimoto T (2012). Therapeutic targeting of the interleukin-6 receptor. *Annual Review of Pharmacology and Toxicology*.

[126] Heymann D, Rousselle A-V (2000). gp130 cytokine family and bone cells. *Cytokine*.

[127] Jay PR, Centrella M, Lorenzo J, Bruce AG, Horowitz MC (1996). Oncostatin-M: a new bone active cytokine that activates osteoblasts and inhibits bone resorption. *Endocrinology*.

[128] Bellido T, Stahl N, Farruggella TJ, Borba V, Yancopoulos GD, Manolagas SC (1996). Detection of receptors for lnterleukin-6, lnterleukin-11, leukemia inhibitory factor, oncostatin M, and ciliary neurotrophic factor in bone marrow stromal/osteoblastic cells. *The Journal of Clinical Investigation*.

[129] Taguchi Y, Yamamoto M, Yamate T (1998). Interleukin-6-type cytokines stimulate mesenchymal progenitor differentiation toward the osteoblastic lineage. *Proceedings of the Association of American Physicians*.

[130] Liu F, Aubin JE, Malaval L (2002). Expression of leukemia inhibitory factor (LIF)/interleukin-6 family cytokines and receptors during in vitro osteogenesis: differential regulation by dexamethasone and LIF. *Bone*.

[131] Malaval L, Liu F, Vernallis AB, Aubin JE (2005). GP130/OSMR is the only LIF/IL-6 family receptor complex to promote osteoblast differentiation of calvaria progenitors. *Journal of Cellular Physiology*.

[132] De Hooge ASK, Van De Loo FAJ, Bennink MB (2002). Adenoviral transfer of murine oncostatin M elicits periosteal bone apposition in knee joints of mice, despite synovial inflammation and up-regulated expression of interleukin-6 and receptor activator of nuclear factor-*κ*B ligand. *American Journal of Pathology*.

[133] Guihard P, Danger Y, Brounais B (2012). Induction of osteogenesis in mesenchymal stem cells by activated monocytes/macrophages depends on oncostatin M signaling. *Stem Cells*.

[134] Walker EC, Poulton IJ, McGregor NE (2012). Sustained RANKL response to parathyroid hormone in oncostatin M receptor-deficient osteoblasts converts anabolic treatment to a catabolic effect in vivo. *Journal of Bone and Mineral Research*.

[135] Fernandes TJ, Hodge JM, Singh PP (2013). Cord blood-derived macrophage-lineage cells rapidly stimulate osteoblastic maturation in mesenchymal stem cells in a glycoprotein-130 dependent manner. *PLoS ONE*.

[136] Nicolaidou V, Wong MM, Redpath AN (2012). Monocytes induce STAT3 activation in human mesenchymal stem cells to promote osteoblast formation. *PLoS ONE*.

[137] Kok S-H, Hong C-Y, Kuo MY-P (2009). Oncostatin M-induced CCL2 transcription in osteoblastic cells is mediated by multiple levels of STAT-1 and STAT-3 signaling: an implication for the pathogenesis of arthritis. *Arthritis and Rheumatism*.

[138] Richards CD, Langdon C, Deschamps P, Pennica D, Shaughnessy SG (2000). Stimulation of osteoclast differentiation in vitro by mouse oncostatin M, leukaemia inhibitory factor, cardiotrophin-1 and interleukin 6: synergy with dexamethasone. *Cytokine*.

[139] Palmqvist P, Persson E, Conaway HH, Lerner UH (2002). IL-6, leukemia inhibitory factor, and oncostatin M stimulate bone resorption and regulate the expression of receptor activator of NF-*κ*B ligand, osteoprotegerin, and receptor activator of NF-*κ*B in mouse calvariae. *The Journal of Immunology*.

[140] Kawashima I, Takiguchi Y (1992). Interleukin-11: a novel stroma-derived cytokine. *Cytokine and Growth Factor Reviews*.

[141] Keller DC, Du XX, Srour EF, Hoffman R, Williams DA (1993). Interleukin-11 inhibits adipogenesis and stimulates myelopoiesis in human long-term marrow cultures. *Blood*.

[142] Ohsumi J, Miyadai K, Kawashima I, Sakakibara S, Yamaguchi J, Itoh Y (1994). Regulation of lipoprotein lipase synthesis in 3T3-L1 adipocytes by interleukin-11/adipogenesis inhibitory factor. *Biochemistry and Molecular Biology International*.

[143] Yanai N, Obinata M (2001). Oncostatin M regulates mesenchymal cell differentiation and enhances hematopoietic supportive activity of bone marrow stromal cell lines. *In Vitro Cellular and Developmental Biology*.

[144] Hae YS, Eun SJ, Jung IK, Jin SJ, Jae HK (2007). Oncostatin M promotes osteogenesis and suppresses adipogenic differentiation of human adipose tissue-derived mesenchymal stem cells. *Journal of Cellular Biochemistry*.

[145] Miyaoka Y, Tanaka M, Naiki T, Miyajima A (2006). Oncostatin M inhibits adipogenesis through the RAS/ERK and STAT5 signaling pathways. *The Journal of Biological Chemistry*.

[146] Zvonic S, Baugh JE, Arbour-Reily P, Mynatt RL, Stephens JM (2005). Cross-talk among gp130 cytokines in adipocytes. *The Journal of Biological Chemistry*.

[147] Hamilton AS, Radka SF, Bernstein L (1994). Relationship of serum levels of oncostatin M to AIDS-related and classic Kaposi’s sarcoma. *Journal of Acquired Immune Deficiency Syndromes*.

[148] Wallace PM, MacMaster JF, Rouleau KA (2000). Erratum: regulation of inflammatory responses by oncostatin M. *The Journal of Immunology*.

[149] Amaral MC, Miles S, Kumar G, Nel AE (1993). Oncostatin-M stimulates tyrosine protein phosphorylation in parallel with the activation of p42(MAPK)/ERK-2 in Kaposi’s cells: evidence that this pathway is important in Kaposi cell growth. *The Journal of Clinical Investigation*.

[150] Lacreusette A, Nguyen J-M, Pandolfino M-C (2007). Loss of oncostatin M receptor *β* in metastatic melanoma cells. *Oncogene*.

[151] Komyod W, Böhm M, Metze D, Heinrich PC, Behrmann I (2007). Constitutive suppressor of cytokine signaling 3 expression confers a growth advantage to a human melanoma cell line. *Molecular Cancer Research*.

[152] Lacreusette A, Lartigue A, Nguyen J-M (2008). Relationship between responsiveness of cancer cells to Oncostatin M and/or IL-6 and survival of stage III melanoma patients treated with tumour-infiltrating lymphocytes. *Journal of Pathology*.

[153] Lacreusette A, Barbieux I, Nguyen J-M (2009). Defective activations of STAT3 Ser727 and PKC isoforms lead to oncostatin M resistance in metastatic melanoma. *Journal of Pathology*.

[154] Mori K, Rédini F, Gouin F, Cherrier B, Heymann D (2006). Osteosarcoma: current status of immunotherapy and future trends (Review). *Oncology Reports*.

[155] Chipoy C, Berreur M, Couillaud S (2004). Downregulation of osteoblast markers and induction of the glial fibrillary acidic protein by oncostatin M in osteosarcoma cells require PKC*δ* and STAT3. *Journal of Bone and Mineral Research*.

[156] Chipoy C, Brounais B, Trichet V (2007). Sensitization of osteosarcoma cells to apoptosis by oncostatin M depends on STAT5 and p53. *Oncogene*.

[157] Brounais B, David E, Chipoy C (2009). Long term oncostatin M treatment induces an osteocyte-like differentiation on osteosarcoma and calvaria cells. *Bone*.

[158] Brounais B, Chipoy C, Mori K (2008). Oncostatin M induces bone loss and sensitizes rat osteosarcoma to the antitumor effect of midostaurin in vivo. *Clinical Cancer Research*.

[159] Fossey SL, Bear MD, Kisseberth WC, Pennell M, London CA (2011). Oncostatin M promotes STAT3 activation, VEGF production, and invasion in osteosarcoma cell lines. *BMC Cancer*.

[160] Douglas AM, Goss GA, Sutherland RL (1997). Expression and function of members of the cytokine receptor superfamily on breast cancer cells. *Oncogene*.

[161] García-Tuñón I, Ricote M, Ruiz A, Fraile B, Paniagua R, Royuela M (2008). OSM, LIF, its receptors, and its relationship with the malignance in human breast carcinoma (in situ and in infiltrative). *Cancer Investigation*.

[162] West NR, Murphy LC, Watson PH (2012). Oncostatin M suppresses oestrogen receptor-alpha expression and is associated with poor outcome in human breast cancer. *Endocrine-Related Cancer*.

[163] Li C, Zhang F, Lin M, Liu J (2004). Induction of S100A9 gene expression by cytokine oncostatin M in breast cancer cells through the STAT3 signaling cascade. *Breast Cancer Research and Treatment*.

[164] West NR, Watson PH (2010). S100A7 (psoriasin) is induced by the proinflammatory cytokines oncostatin-M and interleukin-6 in human breast cancer. *Oncogene*.

[165] Holzer RG, Ryan RE, Tommack M, Schlekeway E, Jorcyk CL (2004). Oncostatin M stimulates the detachment of a reservoir of invasive mammary carcinoma cells: role of cyclooxygenase-2. *Clinical and Experimental Metastasis*.

[166] Jorcyk CL, Holzer RG, Ryan RE (2006). Oncostatin M induces cell detachment and enhances the metastatic capacity of T-47D human breast carcinoma cells. *Cytokine*.

[167] Snyder M, Huang X-Y, Zhang JJ (2011). Signal Transducers and Activators of Transcription 3 (STAT3) directly regulates cytokine-induced fascin expression and is required for breast cancer cell migration. *The Journal of Biological Chemistry*.

[168] Kan CE, Cipriano R, Jackson MW (2011). c-MYC functions as a molecular switch to alter the response of human mammary epithelial cells to oncostatin M. *Cancer Research*.

[169] David E, Guihard P, Brounais B (2011). Direct anti-cancer effect of oncostatin M on chondrosarcoma. *International Journal of Cancer*.

[170] David E, Tirode F, Baud'huin M (2012). Oncostatin M is a growth factor for Ewing sarcoma. *American Journal of Pathology*.

[171] West NR, Murray JI, Watson PH (2013). Oncostatin-M promotes phenotypic changes associated with mesenchymal and stem cell-like differentiation in breast cancer. *Oncogene*.

[172] Guo L, Chen C, Shi M (2013). Stat3-coordinated Lin-28-let-7-HMGA2 and miR-200-ZEB1 circuits initiate and maintain oncostatin M-driven epithelial-mesenchymal transition. *Oncogene*.

[173] Li Q, Zhu J, Sun F, Liu L, Liu X, Yue Y (2011). Oncostatin M promotes proliferation of ovarian cancer cells through signal transducer and activator of transcription 3. *International Journal of Molecular Medicine*.

[174] Ng G, Winder D, Muralidhar B (2007). Gain and overexpression of the oncostatin M receptor occur frequently in cervical squamous cell carcinoma and are associated with adverse clinical outcome. *Journal of Pathology*.

[175] Winder DM, Chattopadhyay A, Muralidhar B (2011). Overexpression of the oncostatin M receptor in cervical squamous cell carcinoma cells is associated with a pro-angiogenic phenotype and increased cell motility and invasiveness. *Journal of Pathology*.

[176] Krona A, Järnum S, Salford LG, Widegren B, Aman P (2005). Oncostatin M signaling in human glioma cell lines. *Oncology reports*.

[177] Chen S-H, Gillespie GY, Benveniste EN (2006). Divergent effects of oncostatin M on astroglioma cells: Influence on cell proliferation, invasion, and expression of matrix metalloproteinases. *GLIA*.

[178] Godoy-Tundidor S, Cavarretta ITR, Fuchs D (2005). Interleukin-6 and Oncostatin M stimulation of proliferation of prostate cancer 22Rv1 cells through the signaling pathways of p38 mitogen-activated protein kinase and phosphatidylinositol 3-kinase. *Prostate*.

[179] Weiss TW, Simak R, Kaun C (2011). Oncostatin M and IL-6 induce u-PA and VEGF in prostate cancer cells and correlate in vivo. *Anticancer Research*.

[180] Royuela M, Ricote M, Parsons MS, García-Tuñón I, Paniagua R, De Miguel MP (2004). Immunohistochemical analysis of the IL-6 family of cytokines and their receptors in benign, hyperplasic and malignant human prostate. *Journal of Pathology*.

[181] Smith DA, Kiba A, Zong Y, Witte ON (2013). Interleukin-6 and oncostatin-M synergize with the PI3K/AKT pathway to promote aggressive prostate malignancy in mouse and human tissues. *Molecular Cancer Research*.

[182] Loewen GM, Tracy E, Blanchard F (2005). Transformation of human bronchial epithelial cells alters responsiveness to inflammatory cytokines. *BMC Cancer*.

[183] Argast GM, Mercado P, Mulford IJ (2010). Cooperative signaling between oncostatin M, hepatocyte growth factor and transforming growth factor-*β* enhances epithelial to mesenchymal transition in lung and pancreatic tumor models. *Cells Tissues Organs*.

[184] Wang ML, Pan CM, Chiou SH (2012). Oncostatin m modulates the mesenchymal-epithelial transition of lung adenocarcinoma cells by a mesenchymal stem cell-mediated paracrine effect. *Cancer Research*.

[185] Chen DR, Chu C-Y, Chen C-Y (2008). Expression of short-form oncostatin M receptor as a decoy receptor in lung adenocarcinomas. *Journal of Pathology*.

[186] Kim MS, Louwagie J, Carvalho B (2009). Promoter DNA methylation of Oncostatin M receptor-*β* as a novel diagnostic and therapeutic marker in colon cancer. *PLoS ONE*.

[187] Deng G, Kakar S, Okudiara K, Choi E, Sleisenger MH, Kim YS (2009). Unique methylation pattern of oncostatin M receptor gene in cancers of colorectum and other digestive organs. *Clinical Cancer Research*.

[188] Hibi K, Goto T, Sakuraba K (2011). Methylation of OSMR gene is frequently observed in non-invasive colorectal cancer. *Anticancer Research*.

[189] Kausar T, Sharma R, Hasan MR (2011). Overexpression of a splice variant of oncostatin M receptor beta in human esophageal squamous carcinoma. *Cellular Oncology*.

[190] Hong IK, Eun YG, Chung DH, Kwon KH, Kim DY (2011). Association of the oncostatin M receptor gene polymorphisms with papillary thyroid cancer in the Korean population. *Clinical and Experimental Otorhinolaryngology*.

[191] Engels B, Rowley DA, Schreiber H (2012). Targeting stroma to treat cancers. *Seminars in Cancer Biology*.

[192] Räsänen K, Vaheri A (2010). Activation of fibroblasts in cancer stroma. *Experimental Cell Research*.

[193] Pietras K, Östman A (2010). Hallmarks of cancer: interactions with the tumor stroma. *Experimental Cell Research*.

[194] Queen MM, Ryan RE, Holzer RG, Keller-Peck CR, Jorcyk CL (2005). Breast cancer cells stimulate neutrophils to produce oncostatin M: potential implications for tumor progression. *Cancer Research*.

[195] Vlaicu P, Mertins P, Mayr T (2013). Monocytes/macrophages support mammary tumor invasivity by co-secreting lineage-specific EGFR ligands and a STAT3 activator. *BMC Cancer*.

[196] Bolin C, Tawara K, Sutherland C (2012). Oncostatin m promotes mammary tumor metastasis to bone and osteolytic bone degradation. *Genes Cancer*.

[197] Benson DD, Meng X, Fullerton DA (2012). Activation state of stromal inflammatory cells in murine metastatic pancreatic adenocarcinoma. *American Journal of Physiology*.

[198] Ruprecht K, Kuhlmann T, Seif F (2001). Effects of oncostatin M on human cerebral endothelial cells and expression in inflammatory brain lesions. *Journal of Neuropathology and Experimental Neurology*.

[199] Van Wagoner NJ, Choi C, Repovic P, Benveniste EN (2000). Oncostatin M regulation of interleukin-6 expression in astrocytes: biphasic regulation involving the mitogen-activated protein kinases ERK1/2 and p38. *Journal of Neurochemistry*.

[200] Repovic P, Mi K, Benveniste EN (2003). Oncostatin M enhances the expression of prostaglandin E2 and cyclooxygenase-2 in astrocytes: synergy with interleukin-1*β*, tumor necrosis factor-*α*, and bacterial lipopolysaccharide. *Glia*.

[201] Repovic P, Fears CY, Gladson CL, Benveniste EN (2003). Oncostatin-M induction of vascular endothelial growth factor expression in astroglioma cells. *Oncogene*.

[202] Krona A, Åman P, Örndal C, Josefsson A (2007). Oncostatin M-induced genes in human astrocytomas. *International Journal of Oncology*.

[203] Glezer I, Rivest S (2010). Oncostatin M is a novel glucocorticoid-dependent neuroinflammatory factor that enhances oligodendrocyte precursor cell activity in demyelinated sites. *Brain, Behavior, and Immunity*.

[204] Beatus P, Jhaveri DJ, Walker TL (2011). Oncostatin M regulates neural precursor activity in the adult brain. *Developmental Neurobiology*.

[205] Kang HJ, Kang JS, Lee SH (2005). Upregulation of oncostatin M in allergic rhinitis. *Laryngoscope*.

[206] Simpson JL, Baines KJ, Boyle MJ, Scott RJ, Gibson PG (2009). Oncostatin m (osm) is increased in asthma with incompletely reversible airflow obstruction. *Experimental Lung Research*.

[207] Mozaffarian A, Brewer AW, Trueblood ES (2008). Mechanisms of oncostatin M-induced pulmonary inflammation and fibrosis. *The Journal of Immunology*.

[208] Luzina IG, Atamas SP, Wise R (2003). Occurrence of an activated, profibrotic pattern of gene expression in lung CD8^+^ T cells from scleroderma patients. *Arthritis and Rheumatism*.

[209] Baines KJ, Simpson JL, Gibson PG (2011). Innate immune responses are increased in chronic obstructive pulmonary disease. *PLoS ONE*.

[210] Elias JA, Lee CG, Zheng T, Ma B, Homer RJ, Zhu Z (2003). New insights into the pathogenesis of asthma. *The Journal of Clinical Investigation*.

[211] Holgate ST (2008). Pathogenesis of asthma. *Clinical and Experimental Allergy*.

[212] Wynn TA (2008). Cellular and molecular mechanisms of fibrosis. *Journal of Pathology*.

[213] Wynn TA (2007). Common and unique mechanisms regulate fibrosis in various fibroproliferative diseases. *The Journal of Clinical Investigation*.

[214] Wynn TA, Ramalingam TR (2012). Mechanisms of fibrosis: therapeutic translation for fibrotic disease. *Nature Medicine*.

[215] DiCosmo BF, Geba GP, Picarella D (1994). Airway epithelial cell expression of interleukin-6 in transgenic mice. Uncoupling of airway inflammation and bronchial hyperreactivity. *The Journal of Clinical Investigation*.

[216] Kuhn C, Homer RJ, Zhu Z (2000). Airway hyperresponsiveness and airway obstruction in transgenic mice: morphologic correlates in mice overexpressing interleukin (IL)-11 and IL-6 in the lung. *American Journal of Respiratory Cell and Molecular Biology*.

[217] Tang W, Geba GP, Zheng T (1996). Targeted expression of IL-11 in the murine airway causes lymphocytic inflammation, bronchial remodeling, and airways obstruction. *The Journal of Clinical Investigation*.

[218] Langdon C, Kerr C, Tong L, Richards CD (2003). Oncostatin M regulates eotaxin expression in fibroblasts and eosinophilic inflammation in C57BL/6 mice. *The Journal of Immunology*.

[219] Xing Z, Braciak T, Jordana M, Croitoru K, Graham FL, Gauldie J (1994). Adenovirus-mediated cytokine gene transfer at tissue sites: overexpression of IL-6 induces lymphocytic hyperplasia in the lung. *The Journal of Immunology*.

[220] Xing Z, Braciak T, Chong D, Feng X, Schroeder J-A, Gauldie J (1999). Adenoviral-mediated gene transfer of interleukin-6 in rat lung enhances antiviral immunoglobulin A and G responses in distinct tissue compartments. *Biochemical and Biophysical Research Communications*.

[221] Richards CD, Kerr C, Tong L, Langdon C (2002). Modulation of extracellular matrix using adenovirus vectors. *Biochemical Society Transactions*.

[222] Lahiri T, Laporte JD, Moore PE, Panettieri RA, Shore SA (2001). Interleukin-6 family cytokines: signaling and effects in human airway smooth muscle cells. *American Journal of Physiology*.

[223] Moore PE, Church TL, Chism DD, Panettieri RA, Shore SA (2002). IL-13 and IL-4 cause eotaxin release in human airway smooth muscle cells: a role for ERK. *American Journal of Physiology*.

[224] Faffe DS, Flynt L, Mellema M (2005). Oncostatin M causes eotaxin-1 release from airway smooth muscle: synergy with IL-4 and IL-13. *Journal of Allergy and Clinical Immunology*.

[225] Faffe DS, Flynt L, Mellema M (2005). Oncostatin M causes VEGF release from human airway smooth muscle: synergy with IL-1*β*. *American Journal of Physiology*.

[226] Richards CD, Agro A (1994). Interaction between Oncostatin M, interleukin 1 and prostaglandin E2 in induction of IL-6 expression in human fibroblasts. *Cytokine*.

[227] Scaffidi AK, Mutsaers SE, Moodley YP (2002). Oncostatin M stimulates proliferation, induces collagen production and inhibits apoptosis of human lung fibroblasts. *British Journal of Pharmacology*.

[228] O’Hara KA, Kedda M-A, Thompson PJ, Knight DA (2003). Oncostatin M: an interleukin-6-like cytokine relevant to airway remodelling and the pathogenesis of asthma. *Clinical and Experimental Allergy*.

[229] Cohen M, Marchand-Adam S, Lecon-Malas V (2006). HGF synthesis in human lung fibroblasts is regulated by oncostatin M. *American Journal of Physiology*.

[230] Fritz DK, Kerr C, Botelho F, Stampfli M, Richards CD (2009). Oncostatin M (OSM) primes IL-13- and IL-4-induced eotaxin responses in fibroblasts: regulation of the type-II IL-4 receptor chains IL-4R*α* and IL-13R*α*1. *Experimental Cell Research*.

[231] Smyth DC, Kerr C, Li Y, Tang D, Richards CD (2008). Oncostatin M induction of eotaxin-1 expression requires the convergence of PI3′K and ERK1/2 MAPK signal transduction pathways. *Cellular Signalling*.

[232] Nagahama KY, Togo S, Holz O (2013). Oncostatin M modulates fibroblast function via STAT3. *American Journal of Respiratory Cell and Molecular Biology*.

[233] Prele CM, Yao E, O'Donoghue RJ, Mutsaers SE, Knight DA (2012). STAT3: a central mediator of pulmonary fibrosis?. *Proceedings of the American Thoracic Society*.

[234] O'Donoghue RJ, Knight DA, Richards CD (2012). Genetic partitioning of interleukin-6 signalling in mice dissociates Stat3 from Smad3-mediated lung fibrosis. *EMBO Molecular Medicine*.

[235] Dudas PL, de GL, Lundblad LK, Knight DA (2012). The role of the epithelium in chronic inflammatory airway disease. *Pulmonary Pharmacology & Therapeutics*.

[236] Sallenave J-M, Tremblay GM, Gauldie J, Richards CD (1997). Oncostatin M, but not interleukin-6 or leukemia inhibitory factor, stimulates expression of alpha1-proteinase inhibitor in A549 human alveolar epithelial cells. *Journal of Interferon and Cytokine Research*.

[237] McCormick C, Freshney RI (2000). Activity of growth factors in the IL-6 group in the differentiation of human lung adenocarcinoma. *British Journal of Cancer*.

[238] Knight DA, Asokananthan N, Watkins DN, Misso NLA, Thompson PJ, Stewart GA (2000). Oncostatin M synergises with house dust mite proteases to induce the production of PGE2 from cultured lung epithelial cells. *British Journal of Pharmacology*.

[239] Tomita T, Yamada A, Miyakoshi M (2009). Oncostatin M regulates secretoglobin 3A1 and 3A2 expression in a bidirectional manner. *American Journal of Respiratory Cell and Molecular Biology*.

[240] Nightingale J, Patel S, Suzuki N (2004). Oncostatin M, a cytokine released by activated mononuclear cells, induces epithelial cell-myofibroblast transdifferentiation via Jak/Stat pathway activation. *Journal of the American Society of Nephrology*.

[241] Sarközi R, Hauser C, Noppert S-J (2011). Oncostatin M is a novel inhibitor of TGF-*β*1-induced matricellular protein expression. *American Journal of Physiology*.

[242] Elbjeirami WM, Truong LD, Tawil A (2010). Early differential expression of oncostatin M in obstructive nephropathy. *Journal of Interferon and Cytokine Research*.

[243] Baumann H, Wang Y, Richards CD, Jones CA, Black TA, Gross KW (2000). Endotoxin-induced renal inflammatory response: oncostatin M as a major mediator of suppressed renin expression. *The Journal of Biological Chemistry*.

[244] Duncan MR, Hasan A, Berman B (1995). Oncostatin M stimulates collagen and glycosaminoglycan production by cultured normal dermal fibroblasts: insensitivity of sclerodermal and keloidal fibroblasts. *The Journal of Investigative Dermatology*.

[245] Ihn H, LeRoy EC, Trojanowska M (1997). Oncostatin M stimulates transcription of the human *α*2(I) collagen gene via the Sp1/Sp3-binding site. *The Journal of Biological Chemistry*.

[246] Ihn H, Tamaki K (2000). Oncostatin M stimulates the growth of dermal fibroblasts via a mitogen- activated protein kinase-dependent pathway. *The Journal of Immunology*.

[247] Hintzen C, Haan C, Tuckermann JP, Heinrich PC, Hermanns HM (2008). Oncostatin M-induced and constitutive activation of the JAK2/STAT5/CIS pathway suppresses CCL1, but Not CCL7 and CCL8, chemokine expression. *The Journal of Immunology*.

[248] Hasegawa M, Sato S, Ihn H, Takehara K (1999). Enhanced production of interleukin-6 (IL-6), oncostatin M and soluble IL-6 receptor by cultured peripheral blood mononuclear cells from patients with systemic sclerosis. *Rheumatology*.

[249] Finelt N, Gazel A, Gorelick S, Blumenberg M (2005). Transcriptional responses of human epidermal keratinocytes to Oncostatin-M. *Cytokine*.

[250] Boniface K, Diveu C, Morel F (2007). Oncostatin M secreted by skin infiltrating T lymphocytes is a potent keratinocyte activator involved in skin inflammation. *The Journal of Immunology*.

[251] Guilloteau K, Paris I, Pedretti N (2010). Skin inflammation induced by the synergistic action of IL-17A, IL-22, oncostatin M, IL-1*α*, and TNF-*α* recapitulates some features of psoriasis. *The Journal of Immunology*.

[252] Giot JP, Paris I, Levillain P (2013). Involvement of IL-1 and oncostatin M in acanthosis associated with hypertensive leg ulcer. *The American Journal of Pathology*.

[253] Sonkoly E, Muller A, Lauerma AI (2006). IL-31: a new link between T cells and pruritus in atopic skin inflammation. *Journal of Allergy and Clinical Immunology*.

[254] Boisvert WA (2004). Modulation of atherogenesis by chemokines. *Trends in Cardiovascular Medicine*.

[255] Sheikine Y, Hansson GK (2004). Chemokines and atherosclerosis. *Annals of Medicine*.

[256] Charo IF, Taubman MB (2004). Chemokines in the pathogenesis of vascular disease. *Circulation Research*.

[257] Curtiss LK, Kubo N, Schiller NK, Boisvert WA (2000). Participation of innate and acquired immunity in atherosclerosis. *Immunologic Research*.

[258] Huber SA, Sakkinen P, Conze D, Hardin N, Tracy R (1999). Interleukin-6 exacerbates early atherosclerosis in mice. *Arteriosclerosis, Thrombosis, and Vascular Biology*.

[259] Madan M, Bishayi B, Hoge M, Amar S (2008). Atheroprotective role of interleukin-6 in diet- and/or pathogen-associated atherosclerosis using an ApoE heterozygote murine model. *Atherosclerosis*.

[260] Boyle JJ (2005). Macrophage activation in atherosclerosis: pathogenesis and pharmacology of plaque rupture. *Current Vascular Pharmacology*.

[261] Boyle JJ, Weissberg PL, Bennett MR (2003). Tumor necrosis factor-*α* promotes macrophage-induced vascular smooth muscle cell apoptosis by direct and autocrine mechanisms. *Arteriosclerosis, Thrombosis, and Vascular Biology*.

[262] Brown TJ, Rowe JM, Liu J, Shoyab M (1991). Regulation of IL-6 expression by oncostatin M. *The Journal of Immunology*.

[263] Brown TJ, Liu J, Brashem-Stein C, Shoyab M (1993). Regulation of granulocyte colony-stimulating factor and granulocyte- macrophage colony-stimulating factor expression by oncostatin M. *Blood*.

[264] Modur V, Feldhaus MJ, Weyrich AS (1997). Oncostatin M is a proinflammatory mediator: in vivo effects correlate with endothelial cell expression of inflammatory cytokines and adhesion molecules. *The Journal of Clinical Investigation*.

[265] Yao L, Pan J, Setiadi H, Patel KD, McEver RP (1996). Interleukin 4 or oncostatin M induces a prolonged increase in P-selectin mRNA and protein in human endothelial cells. *Journal of Experimental Medicine*.

[266] Kerfoot SM, Raharjo E, Ho M (2001). Exclusive neutrophil recruitment with oncostatin M in a human system. *American Journal of Pathology*.

[267] Pourtau J, Mirshahi F, Li H (1999). Cyclooxygenase-2 activity is necessary for the angiogenic properties of oncostatin M. *FEBS Letters*.

[268] Rychli K, Kaun C, Hohensinner PJ (2010). The inflammatory mediator oncostatin M induces angiopoietin 2 expression in endothelial cells in vitro and in vivo. *Journal of Thrombosis and Haemostasis*.

[269] Vasse M, Pourtau J, Trochon V (1999). Oncostatin M induces angiogenesis in vitro and in vivo. *Arteriosclerosis, Thrombosis, and Vascular Biology*.

[270] Grove RI, Eberhardt C, Abid S (1993). Oncostatin M is a mitogen for rabbit vascular smooth muscle cells. *Proceedings of the National Academy of Sciences of the United States of America*.

[271] Bernard C, Merval R, Lebret M (1999). Oncostatin M induces interleukin-6 and cyclooxygenase-2 expression in human vascular smooth muscle cells: synergy with interleukin-1*β*. *Circulation Research*.

[272] Demyanets S, Kaun C, Rychli K (2011). Oncostatin M-enhanced vascular endothelial growth factor expression in human vascular smooth muscle cells involves PI3K-, p38 MAPK-, Erk1/2- and STAT1/STAT3-dependent pathways and is attenuated by interferon-*γ*. *Basic Research in Cardiology*.

[273] Albasanz-Puig A, Murray J, Preusch M (2011). Oncostatin M is expressed in atherosclerotic lesions: a role for Oncostatin M in the pathogenesis of atherosclerosis. *Atherosclerosis*.

[274] Macfelda K, Weiss TW, Kaun C (2002). Plasminogen activator inhibitor 1 expression is regulated by the inflammatory mediators interleukin-1*α*, tumor necrosis factor-*α*, transforming growth factor-*β* and oncostatin M in human cardiac myocytes. *Journal of Molecular and Cellular Cardiology*.

[275] Weiss TW, Speidl WS, Kaun C (2003). Glycoprotein 130 ligand oncostatin-M induces expression of vascular endothelial growth factor in human adult cardiac myocytes. *Cardiovascular Research*.

[276] Weiss TW, Kvakan H, Kaun C (2005). The gp130 ligand oncostatin M regulates tissue inhibitor of metalloproteinases-1 through ERK1/2 and p38 in human adult cardiac myocytes and in human adult cardiac fibroblasts: a possible role for the gp130/gp130 ligand system in the modulation of extracellular matrix degradation in the human heart. *Journal of Molecular and Cellular Cardiology*.

[277] Hohensinner PJ, Kaun C, Rychli K (2009). The inflammatory mediator oncostatin M induces stromal derived factor-1 in human adult cardiac cells. *FASEB Journal*.

[278] Kubin T, Pöling J, Kostin S (2011). Oncostatin M is a major mediator of cardiomyocyte dedifferentiation and remodeling. *Cell Stem Cell*.

